# Interleukin‐10 Derived Apoptotic Vesicles Enhance Scarless Skin Healing by Modulating Fibroblast Metabolism and Fibrosis Pathways

**DOI:** 10.1111/cpr.70135

**Published:** 2025-10-12

**Authors:** Yang Zou, Jinglun Zhang, Wenxuan Mao, Shuting Jiang, Cheng Xu, Jiayi Meng, Heng Dong, Yongbin Mou

**Affiliations:** ^1^ Nanjing Stomatological Hospital, Affiliated Hospital of Medical School, Institute of Stomatology Nanjing University Nanjing China

## Abstract

Open skin wounds caused by burns, trauma, or underlying diseases impose substantial clinical challenges and significantly compromise patients' quality of life due to their complex management and high risk of scarring. In this study, we explore the therapeutic potential of apoptotic vesicles derived from interleukin‐10‐treated fibroblasts (IL10_ApoEVs) in promoting cutaneous wound healing and mitigating fibrotic scar formation. Our results demonstrate that IL10_ApoEVs enhance mitochondrial function and oxidative phosphorylation (OXPHOS), while concurrently suppressing glycolytic activity in fibroblasts. Importantly, IL10_ApoEVs markedly inhibit the Hedgehog signalling pathway, a key driver of fibrogenesis in various tissues, as evidenced by the downregulation of Shh and Gli1 expression. This modulation leads to attenuated aberrant extracellular matrix (ECM) deposition and promotes a favourable shift in collagen composition. This is characterized by increased type III collagen and reduced type I collagen, which is indicative of more elastic and functionally integrated tissue remodelling. These findings suggest that IL10_ApoEVs contribute to a regenerative microenvironment that supports scarless or minimally fibrotic healing. Collectively, our work highlights the promising application of IL10_ApoEVs in regenerative medicine and provides mechanistic insights into their dual role in metabolic reprogramming and antifibrotic signalling modulation during tissue repair.

## Graphical abstract

IL10_ApoEVs, generated during IL‐10‐induced fibroblast apoptosis, reprogram fibroblast metabolism and inhibit fibrosis to promote scarless skin healing. By enhancing mitochondrial OXPHOS, suppressing glycolysis, and downregulating Hedgehog signaling, IL10_ApoEVs shift collagen deposition toward regenerative type III collagen, offering a novel strategy for functional skin regeneration.

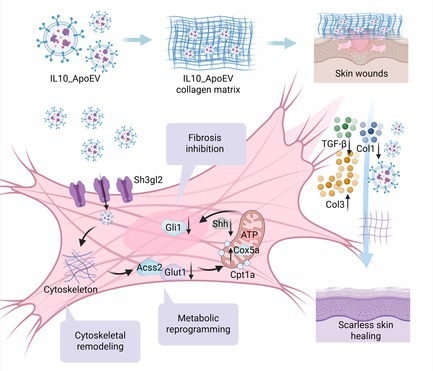

## 1 Introduction

Open skin wounds, whether due to burns, trauma, cancer, or genetic disorders, severely affect quality of life and impose substantial financial and psychological burdens [1, 2]. Following injury, the body initiates repair inherent mechanisms to restore skin structure and function [3]. Although skin grafts are currently the most effective treatment option, they are beset with challenges, including immune rejection, metabolic stressors, skin graft shortage, and high rates of apoptosis in transplanted tissues [4, 5]. Moreover, even under ideal conditions, fibrosis or scar formation is a common outcome [6, 7]. Reconstructing appendage‐bearing skin (i.e., follicles and glands) is a biomedical challenge that has yet to be met [8]. Recent advancements have thus been directed toward developing alternative therapies that decrease reliance on skin grafts and diminish associated complications [9, 10, 11, 12, 13]. These strategies aim to improve patient outcomes by enhancing the physiological integration and functional recovery of injured skin.

Apoptosis, once primarily associated with developmental processes, is now increasingly recognized as a critical regulator of inflammation resolution, wound remodelling, and skin regeneration [14, 15, 16, 17, 18]. During physiological wound healing, apoptosis facilitates the clearance of inflammatory cells and granulation tissue, thereby contributing to reduced scar formation [19, 20, 21]. In contrast, delayed or insufficient apoptosis may impede wound healing and exacerbate fibrotic scarring [22, 23, 24]. Despite these observations, the precise molecular mechanisms by which apoptosis influences scar formation remain inadequately understood. Recent studies have identified apoptotic vesicles (ApoEVs), which are membrane‐bound extracellular vesicles released by apoptotic cells, as active participants in intercellular communication. ApoEVs are efficiently internalized by phagocytes through well‐characterized “find me” and “eat me” signalling pathways [25, 26]. These vesicles encapsulate and deliver bioactive molecules and molecular signatures from their parent cells, thereby modulating the phenotype and function of recipient cells [27]. Notably, ApoEVs have shown promise in transplantation settings, where they reduce immunogenicity and mitigate rejection, thereby improving graft tolerance and survival [28, 29]. Such immunomodulatory properties also position ApoEVs as potential therapeutic agents for tissue regeneration in skin, bone, and muscle repair [30, 31, 32, 33]. Among all extracellular vesicle subtypes, ApoEVs may possess unique advantages due to their distinct cargo profiles, targeted immunomodulatory effects, and efficient uptake mechanisms. These attributes underscore their emerging relevance in wound healing research [34]. Nevertheless, the specific mechanisms by which ApoEVs promote skin regeneration and attenuate scar formation remain largely undefined. Wound healing is orchestrated through a dynamic interplay of rapidly proliferating cells and a cytokine‐rich immune microenvironment, where both pro‐inflammatory and anti‐inflammatory mediators coexist [35, 36]. Importantly, these cytokines act in concert with apoptotic cells and ApoEVs within the wound milieu; however, the nature and consequences of their interactions are still not fully elucidated. A deeper understanding of the cross‐talk between apoptotic processes and cytokine signalling may be essential for devising strategies that optimise skin regeneration while minimizing fibrotic outcomes.

Fibroblasts serve as pivotal regulators in the wound healing cascade, orchestrating extracellular matrix (ECM) synthesis, collagen deposition, and tissue remodelling [37]. However, aberrant activation of fibroblasts frequently leads to pathological scar formation, positioning them as a critical target for therapeutic modulation in regenerative medicine [38]. Interleukin‐10 (IL‐10), a potent anti‐inflammatory cytokine predominantly secreted by macrophages within the wound microenvironment, plays an essential role in orchestrating tissue repair processes [39, 40, 41]. IL‐10 mitigates scarring by inhibiting the release of pro‐inflammatory cytokines, suppressing excessive ECM production, and preventing fibroblast‐to‐myofibroblast transdifferentiation [42]. These mechanisms collectively contribute not only to the attenuation of inflammation but also to the fine‐tuning of tissue remodelling and fibrosis, which are key factors in achieving scarless wound healing. Moreover, rapid IL‐10 production by donor epithelial cells following skin grafting has been shown to enhance graft survival, underscoring its broader immunomodulatory function in transplantation contexts [43, 44, 45]. This suggests that IL‐10 may facilitate immune tolerance and promote tissue integration in allogeneic settings. While the regenerative benefits of ApoEVs derived from untreated cells have been increasingly recognized, the specific functional mechanisms underlying ApoEVs derived from IL‐10‐treated cells remain largely unexplored. In particular, there is growing interest in determining whether IL‐10‐conditioned ApoEVs, especially those generated by fibroblasts, exhibit distinct biological activities that could further enhance wound healing and reduce fibrosis. Elucidating these differential effects may provide novel insights into how anti‐inflammatory cues can be leveraged to optimise extracellular vesicle‐based therapies for tissue regeneration.

**FIGURE 1:**
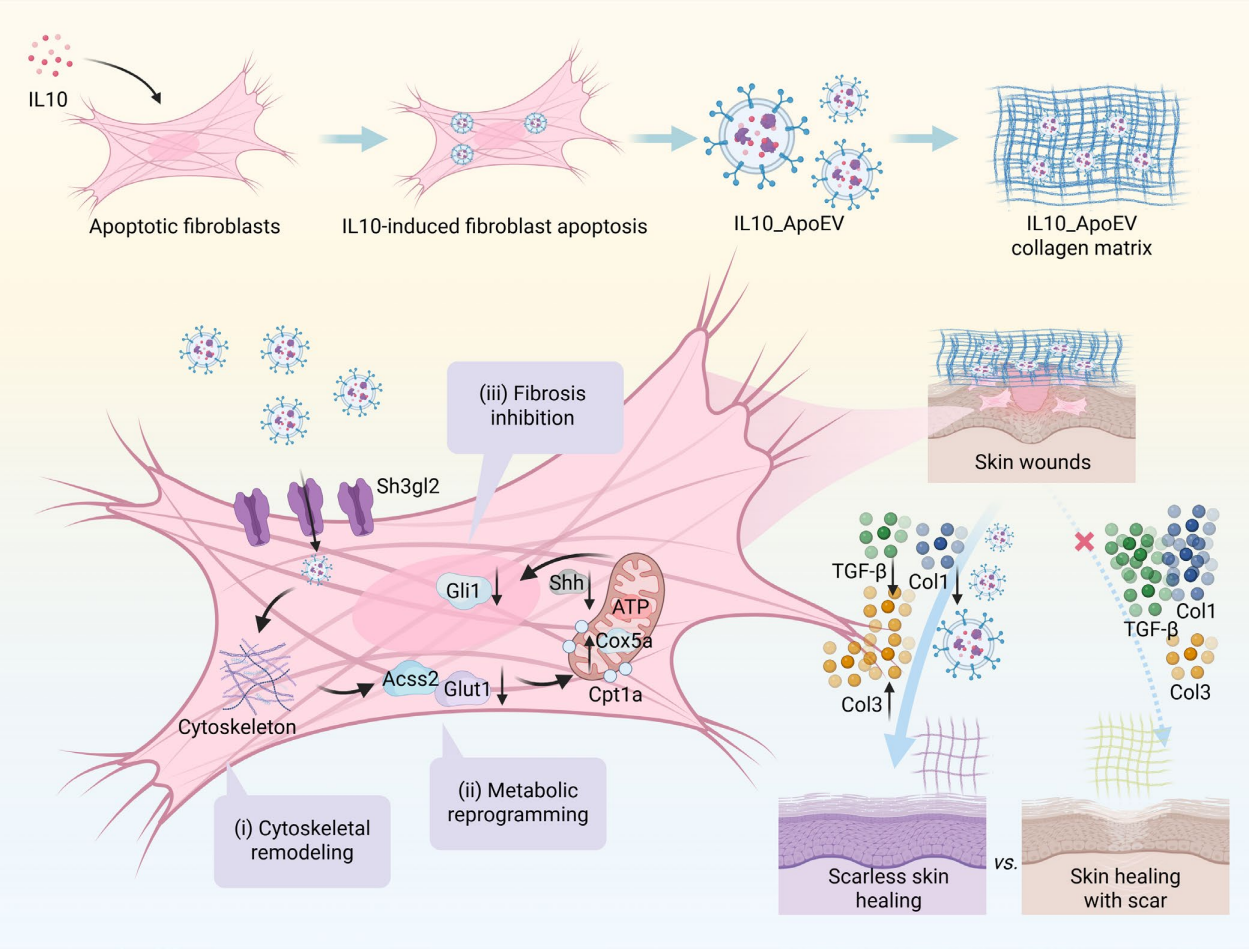
Schematic illustration of IL10_ApoEV‐mediated promotion of scarless healing in skin wounds. IL10_ApoEVs are generated during IL‐10‐induced fibroblast apoptosis. Upon topical application to skin wounds, IL10_ApoEVs are internalized by local fibroblasts through endocytic pathways facilitated by the membrane protein Sh3gl2. Once internalized, these vesicles exert multiple regulatory effects: (I) Cytoskeletal remodelling: IL10_ApoEVs promote reorganization of the Actin cytoskeleton, enhancing fibroblast migration and tissue integration. (ii) Metabolic reprogramming: They suppress glycolysis while promoting mitochondrial OXPHOS, improving energy efficiency for wound repair. (iii) Fibrosis inhibition: IL10_ApoEVs downregulate Hedgehog pathway components (Shh, Gli1), leading to decreased expression of profibrotic markers such as TGF‐β and type I collagen (Col1), while upregulating type III collagen (Col3), a key component in regenerative, scarless tissue architecture. Collectively, these actions reprogram fibroblast function toward regenerative phenotypes and reduce fibrotic scarring, thereby enhancing functional skin regeneration.

In this study, we explored the therapeutic efficacy of apoptotic extracellular vesicles (ApoEVs) derived from IL‐10‐treated fibroblasts (IL10_ApoEVs) in promoting skin wound healing and minimizing scar formation. Our results revealed that IL10_ApoEVs exhibited significantly enhanced uptake by fibroblasts compared to ApoEVs derived from untreated cells. This increased internalization was associated with a metabolic shift characterized by suppressed glycolytic activity and elevated oxidative phosphorylation (OXPHOS). Concurrently, IL10_ApoEVs modulated key fibrotic signalling pathways, notably downregulating the expression of Sonic Hedgehog (Shh) and Gli1. These molecular alterations culminated in improved wound healing outcomes, including reduced fibrotic tissue formation and the regeneration of appendage‐bearing skin structures, indicative of functional tissue repair (Figure 1). Compared to conventional ApoEVs, IL10_ApoEVs demonstrated a superior ability to orchestrate a pro‐regenerative and anti‐fibrotic microenvironment. Moreover, the ability of IL10_ApoEVs to modulate both cellular metabolism and fibrotic signalling underscores the complexity of their biological effects and highlights the therapeutic potential of cytokine‐preconditioned vesicles. These findings provide new mechanistic insights into how immune‐regulatory conditioning can enhance the regenerative properties of ApoEVs, offering a promising strategy to prevent excessive fibrosis and improve the quality of skin repair in wound management.

## 2 Result and Discussion

### 2.1 Preparation and Characterization of ApoEV and IL10_ApoEV

**FIGURE 2:**
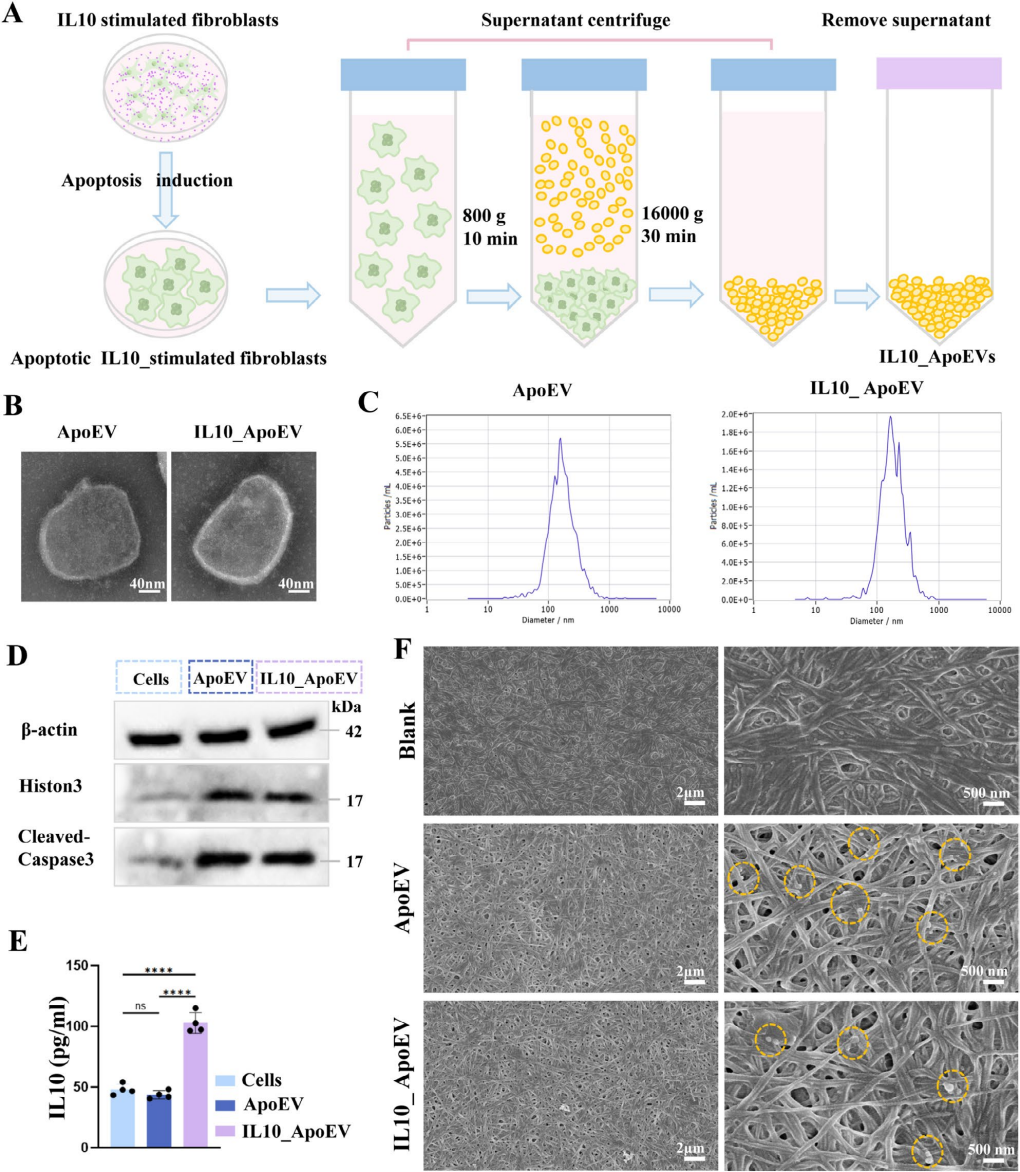
Isolation and characterization of IL10_ApoEVs and ApoEVs. (A) Schematic diagram illustrating the workflow for extraction and purification of ApoEVs and IL10_ApoEVs from fibroblasts, with or without IL‐10 pretreatment. (B) Representative transmission electron microscopy (TEM) images showing the typical spherical morphology and intact membrane structure of both ApoEVs and IL10_ApoEVs. (C) Nanoparticle tracking analysis (NTA) results showing the particle size distribution profiles of the two vesicle populations. (D) Western blot analysis confirming the apoptotic origin of the vesicles via detection of characteristic proteins, Histone H3 and cleaved Caspase‐3. (E) Quantification of IL‐10 content in the vesicles using ELISA, demonstrating significantly elevated levels in IL10_ApoEVs. (F) Scanning electron microscopy (SEM) images of collagen scaffolds loaded with ApoEVs or IL10_ApoEVs, showing effective vesicle embedding and retention within the scaffold microstructure.

In this study, we successfully prepared two types of apoptotic extracellular vesicles: conventional apoptotic vesicles (ApoEV), derived from fibroblasts undergoing apoptosis, and IL10_ApoEV, obtained from fibroblasts pretreated with interleukin‐10 (IL‐10) prior to apoptosis induction (Figure 2A). Transmission electron microscopy (TEM) and nanoparticle tracking analysis (NTA) revealed that both vesicle types exhibited comparable spherical morphology and similar particle size distributions (Figure 2B,C). This suggests that IL‐10 preconditioning does not significantly affect the basic physicochemical properties of ApoEVs. Western blot analysis confirmed the apoptotic origin of the vesicles, as evidenced by the presence of apoptosis‐associated proteins, including histone H3 and cleaved caspase‐3, in both ApoEV and IL10_ApoEV groups (Figure 2D). Importantly, ELISA quantification revealed a markedly higher IL‐10 protein content in the IL10_ApoEVs compared to the control ApoEVs (Figure 2E), indicating successful bioencapsulation of IL‐10. This enrichment may endow IL10_ApoEVs with enhanced anti‐inflammatory and immunomodulatory potential, which is critical for therapeutic applications in wound healing. To evaluate the delivery potential and therapeutic applicability of these vesicles, we embedded them into collagen scaffolds, which are widely used in tissue engineering due to their biocompatibility and structural support. Three scaffold groups were fabricated: blank collagen scaffold, ApoEV‐loaded collagen scaffold, and IL10_ApoEV‐loaded collagen scaffold. Scanning electron microscopy (SEM) confirmed the successful and uniform integration of both ApoEV and IL10_ApoEV into the collagen matrix, with vesicles clearly visible on the fibre surfaces (Figure 2F). These results suggest that collagen scaffolds can serve as efficient delivery platforms for sustained and localized administration of apoptotic vesicles, paving the way for their application in enhancing wound healing and tissue regeneration.

### 2.2 Enhanced Skin Regeneration Properties of Collagen Scaffolds Incorporating IL10_ApoEV

**FIGURE 3:**
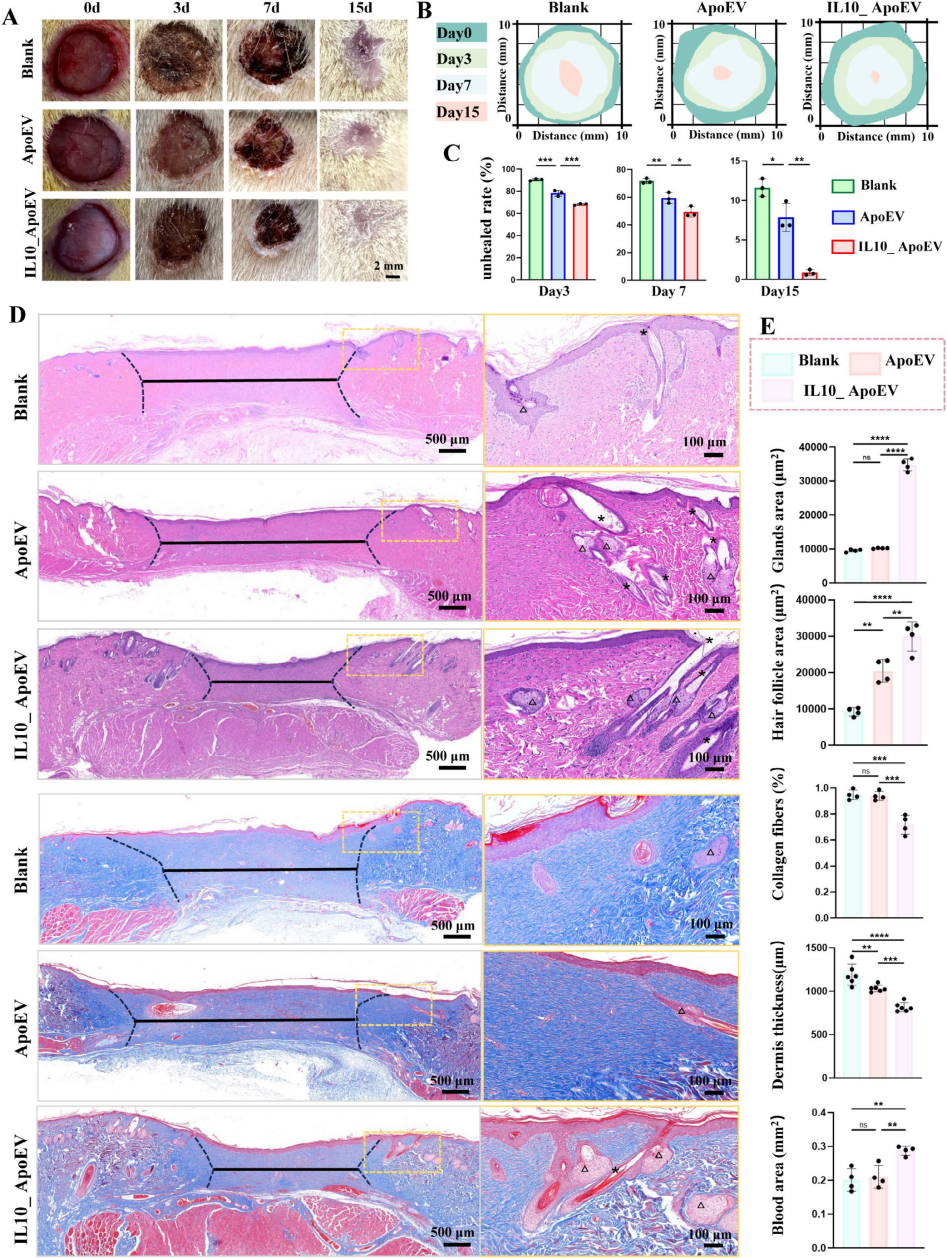
Evaluation of skin wound healing promoted by apoptotic vesicle‐based treatments. (A) Representative photos showing the time course of wound closure across treatment groups. (B) Schematic representation of wound healing progression, illustrating morphological differences between groups. (C) Quantitative analysis of wound closure rates over time. (D) Histological examination of wound sites on day 15 using haematoxylin and eosin (H&E) and Masson's trichrome staining. Key features include wound margins (dashed black lines), wound width (solid black lines), newly formed hair follicles (black asterisks), and sebaceous glands (black triangles). (E) Semi‐quantitative comparison of regenerative parameters among different groups, including glandular area, hair follicle density, collagen fibre deposition, dermal thickness, and neovascularization (blood area).

To evaluate the regenerative efficacy of apoptotic vesicle‐based therapies, three types of collagen scaffolds (Blank, ApoEV‐loaded, and IL10_ApoEV‐loaded) were implanted into full‐thickness dorsal skin defects in rats. Macroscopic observations over a 15‐day period revealed that wounds treated with IL10_ApoEV scaffolds exhibited markedly accelerated closure compared to the ApoEV and Blank groups. By day 15, the IL10_ApoEV group achieved nearly complete epithelial coverage with minimal residual wound area (Figure 3A–C). Histological analysis using haematoxylin and eosin (H&E) and Masson's trichrome staining further demonstrated superior tissue regeneration in the IL10_ApoEV group (Figure 3D). Specifically, this group exhibited enhanced re‐epithelialization, evident by the reconstitution of dermal appendages such as hair follicles and sebaceous glands. Furthermore, the regenerated tissue in this group showed minimal fibrotic features, as reflected by reduced collagen fibre deposition, and maintained a more physiologically relevant dermal thickness. Quantitative assessments corroborated these findings (Figure 3E). IL10_ApoEV treatment significantly increased the areas of newly formed hair follicles and sebaceous glands while reducing collagen density—indicative of decreased scar formation. The group also demonstrated enhanced neovascularization and preservation of blood supply, supporting tissue remodelling and perfusion. These results highlight the potent regenerative capacity of IL10_ApoEVs in promoting scarless healing by restoring both the structural and functional integrity of the skin.

**FIGURE 4:**
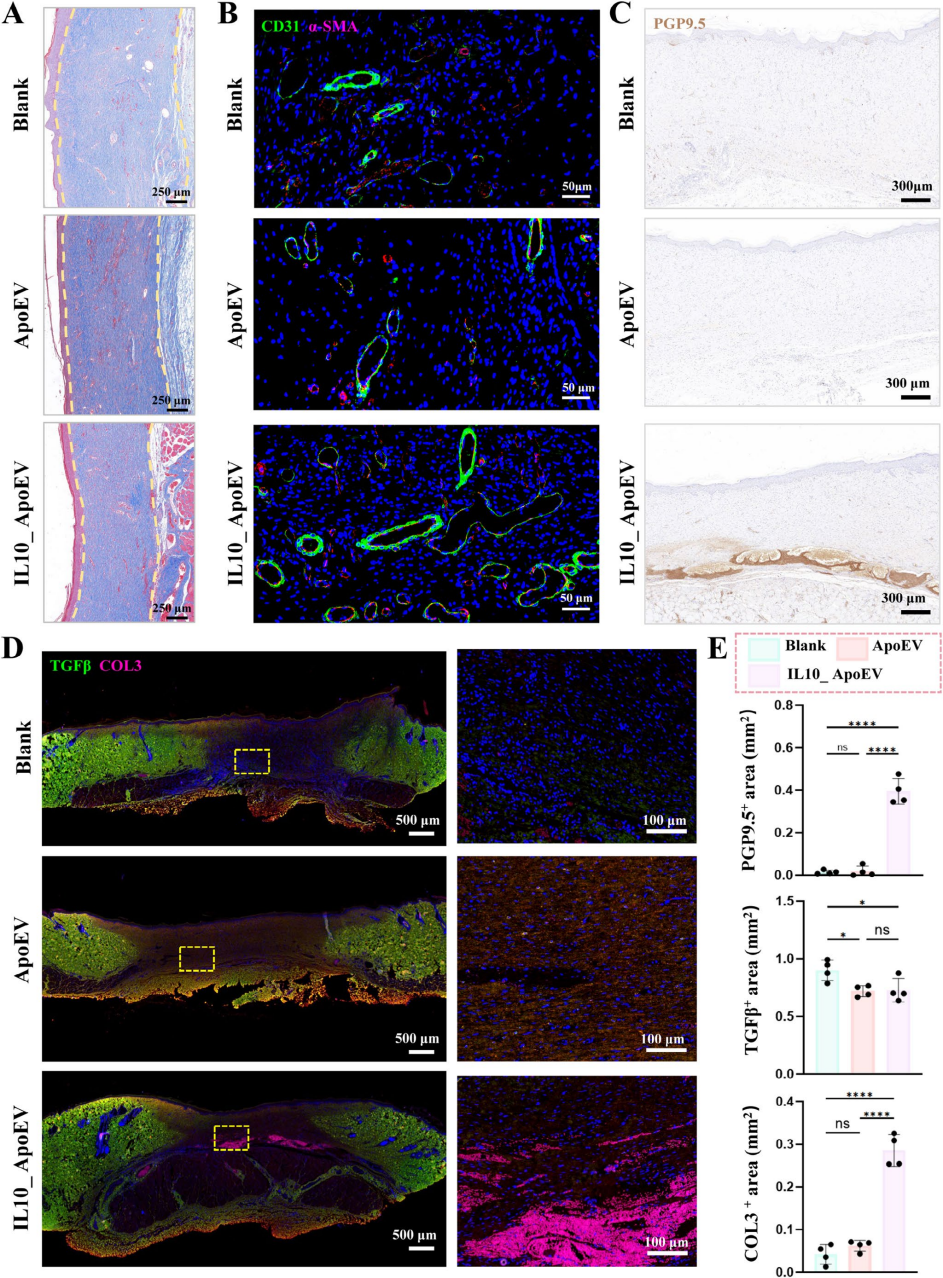
Comparative evaluation of wound healing and regenerative outcomes induced by ApoEV and IL10_ApoEV Treatment. (A) Masson's trichrome staining of local regenerated skin on day 15 post‐wounding reveals dermal architecture and collagen distribution across groups. (B) Immunofluorescence co‐staining of CD31 (green) and α‐SMA (magenta) highlights neovascularization and perivascular myofibroblast activation, respectively. (C) Immunohistochemical staining of PGP9.5. (D) Immunofluorescence co‐staining of TGF‐β (green) and COL3 (magenta) identifies fibrotic signalling and matrix quality. (E) Semi‐quantitative analysis of regenerative markers across treatment groups, including PGP9.5^+^, TGFβ^+^ and COL3^+^ area.

To further elucidate the effects of IL10_ApoEVs on scar formation and regenerative quality, we evaluated tissue samples on day 15 post‐treatment using a combination of histological and immunofluorescence staining. As shown in Figure 4A, Masson's trichrome staining showed better dermal architecture with reduced collagen deposition, indicating attenuated fibrosis. Immunofluorescence staining further confirmed that IL10_ApoEV treatment led to robust neovascularization, as evidenced by increased CD31^+^ and α‐SMA^+^ vessel formation (Figure 4B), and marked reinnervation, with significantly elevated PGP9.5^+^ nerve fibre presence (Figure 4C,E). Most notably, IL10_ApoEVs modulated the fibrotic environment by downregulating TGF‐β, a major pro‐fibrotic cytokine [46], and upregulating type III collagen (COL3) (Figure 4D,E). This is of particular importance, as type III collagen plays a crucial role in early, flexible matrix formation that supports cell migration and angiogenesis, while reducing tissue stiffness and contracture. Unlike type I collagen, which predominates in hypertrophic scars and contributes to tissue rigidity, COL3 facilitates scarless and elastic skin regeneration and transitions in a regulated manner during late remodelling phases. These molecular shifts suggest that IL10_ApoEVs not only accelerate wound closure but actively reprogram the tissue healing trajectory, suppressing fibrotic signalling while promoting a regenerative matrix environment. This multifaceted improvement, including combining vascularization, innervation, reduced fibrosis, and enhanced ECM remodelling, underscores the therapeutic potential of IL10_ApoEVs as precision modulators of the wound microenvironment, capable of supporting functional and cosmetically favourable skin repair.

### 2.3 Enhancement of Endocytosis in Fibroblasts by IL10_ApoEV

**FIGURE 5:**
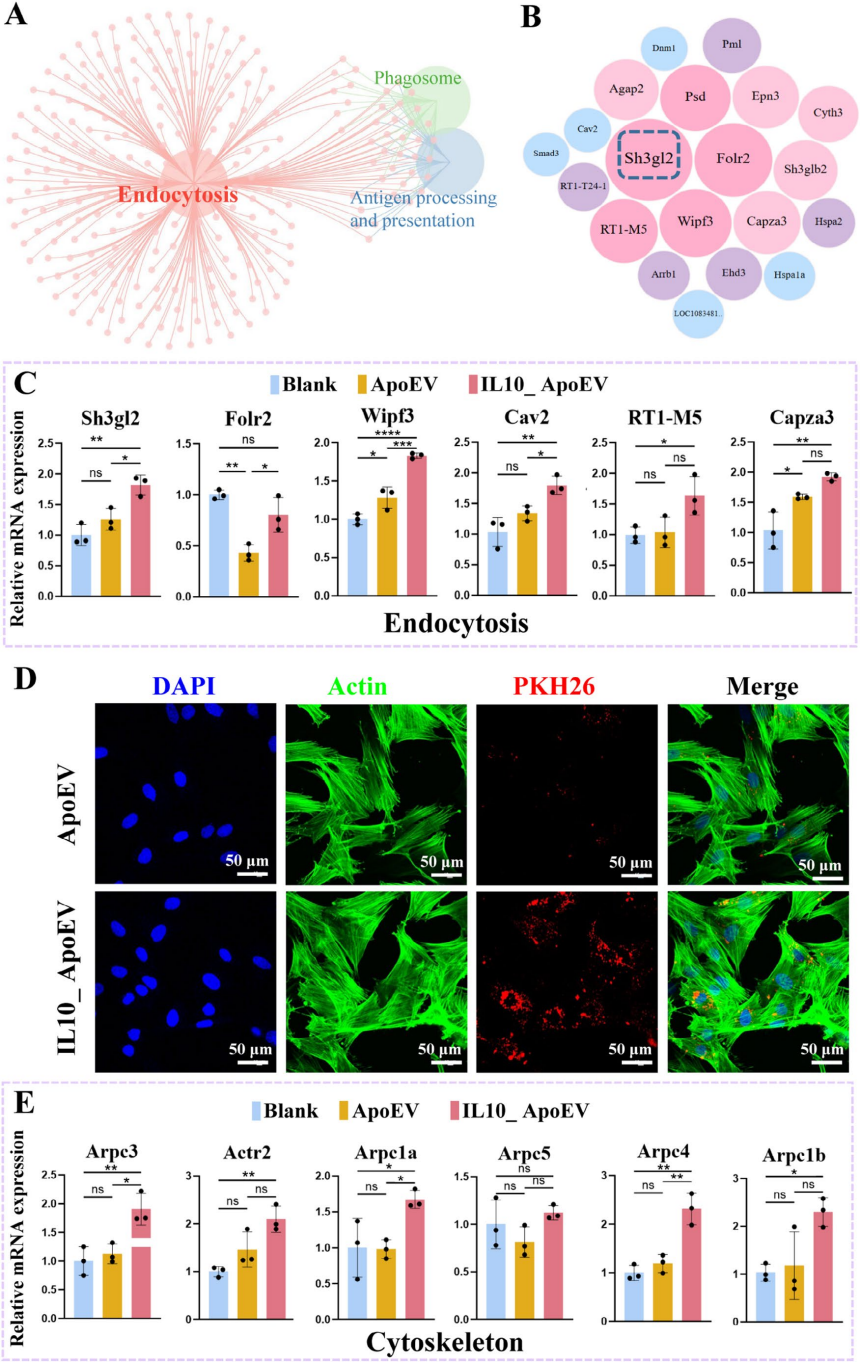
IL10_ApoEV regulates fibroblast glycolytic metabolism through modulation of cytoskeletal organization and endocytotic pathways. (A) KEGG pathway enrichment analysis highlighting the endocytosis pathway significantly affected by IL10_ApoEV treatment. (B) Bubble plot showing differentially expressed genes involved in the endocytosis pathway. (C) RT‐qPCR validation of representative genes associated with endocytosis. (D) Immunofluorescence staining depicting the distribution of endocytosis‐related protein PKH26 (red) and cytoskeleton‐associated protein Actin (green), illustrating enhanced vesicle uptake and cytoskeletal remodelling. (E) RT‐qPCR analysis of cytoskeleton‐related genes, confirming transcriptional alterations induced by IL10_ApoEV.

Fibroblasts are central players in skin tissue regeneration due to their pivotal roles in extracellular matrix production, remodelling, and crosstalk with other cell types during wound repair [47]. Importantly, their capacity to integrate external signals such as extracellular vesicles determines whether healing proceeds toward regenerative repair or pathological scarring. To elucidate how IL10_ApoEV modulates fibroblast activity, we co‐cultured fibroblasts with ApoEV or IL10_ApoEV and performed RNA sequencing to identify differentially expressed genes (DEGs) associated with functional changes. KEGG pathway enrichment revealed a pronounced activation of the endocytosis pathway in the IL10_ApoEV group compared with ApoEV (Figures 5A, S1), indicating that IL10_ApoEV treatment specifically augments vesicle internalization processes. Within this pathway, *Sh3gl2* was identified as the most significantly regulated gene, showing robust upregulation in IL10_ApoEV‐treated fibroblasts (Figure 5B). RT‐qPCR validation further confirmed enhanced expression of *Sh3gl2*, together with *Wipf3* and *Cav2*, all of which are key regulators of endocytosis and vesicular trafficking (Figure 5C). These transcriptional changes translated into functional effects, as demonstrated by immunofluorescence staining. PKH26, a fluorescent dye incorporated into ApoEV membranes, showed markedly higher intracellular signal intensity in the IL10_ApoEV group, confirming more efficient vesicle uptake (Figure 5D). This finding strongly supports the notion that IL10_ApoEV enhances endocytosis at both the molecular and cellular levels. Interestingly, this increase in endocytic activity was closely associated with cytoskeletal remodelling. The actin cytoskeleton provides the structural framework required for vesicle internalization and trafficking. Consistent with this, several cytoskeleton‐associated genes, including *Arpc3*, *Arpc1a*, and *Arpc4*, were upregulated following IL10_ApoEV exposure (Figure 5E). These genes are integral components of the Arp2/3 complex, which mediates actin nucleation and branching, thereby facilitating dynamic cytoskeletal rearrangements required for vesicle engulfment. Together, these results suggest that IL10_ApoEV enhances fibroblast endocytosis through a coordinated regulation of both endocytosis‐specific genes (*Sh3gl2*, *Wipf3*, *Cav2*) and cytoskeleton‐related genes (*Arpc3*, *Arpc1a*, *Arpc4*). By amplifying endocytotic activity and remodelling actin dynamics, IL10_ApoEV likely enhances fibroblast responsiveness to extracellular cues, promoting scar‐free tissue repair and functional skin regeneration.

The process of endocytosis is intricately linked with cellular metabolism, as evidenced by enrichment analysis of DEGs which revealed significant enrichment in the glycolysis pathway (Figure S1). Network analysis further illustrated the close interaction between genes governing endocytosis and cytoskeletal dynamics with those regulating glycolysis, suggesting that IL10_ApoEV orchestrates a coordinated regulation of vesicle trafficking and metabolic reprogramming (Figure S2A). Within this network, multiple glycolytic enzymes, including *Pkm*, *Ldha*, *Eno1*, *Pgam1*, *Aldoa*, and *Pgk1*, were differentially expressed, reflecting a systematic shift in fibroblast energy metabolism. Heatmap visualization of the top glycolytic genes confirmed this trend, with several showing consistent downregulation upon IL10_ApoEV treatment (Figure S2B). Among them, Acss2 stood out as the most prominently downregulated gene, a finding further validated by RT‐qPCR analysis (Figure S2C). Given that *ACSS2* facilitates the conversion of acetate into acetyl‐CoA, its downregulation suggests a metabolic reorientation away from acetate‐dependent acetyl‐CoA generation, potentially limiting fibroblast hyperactivation and fibrosis. Conversely, moderated expression of other glycolytic genes indicates a fine‐tuned metabolic state that may support the energetic and biosynthetic needs of fibroblasts. Thus, IL10_ApoEV not only enhances endocytosis but also reshapes glycolytic metabolism, establishing a cellular environment conducive to controlled regeneration and reduced scar formation.

### 2.4 Enhancement of Mitochondrial Function and OXPHOs by IL10_ApoEV in Fibroblasts

**FIGURE 6:**
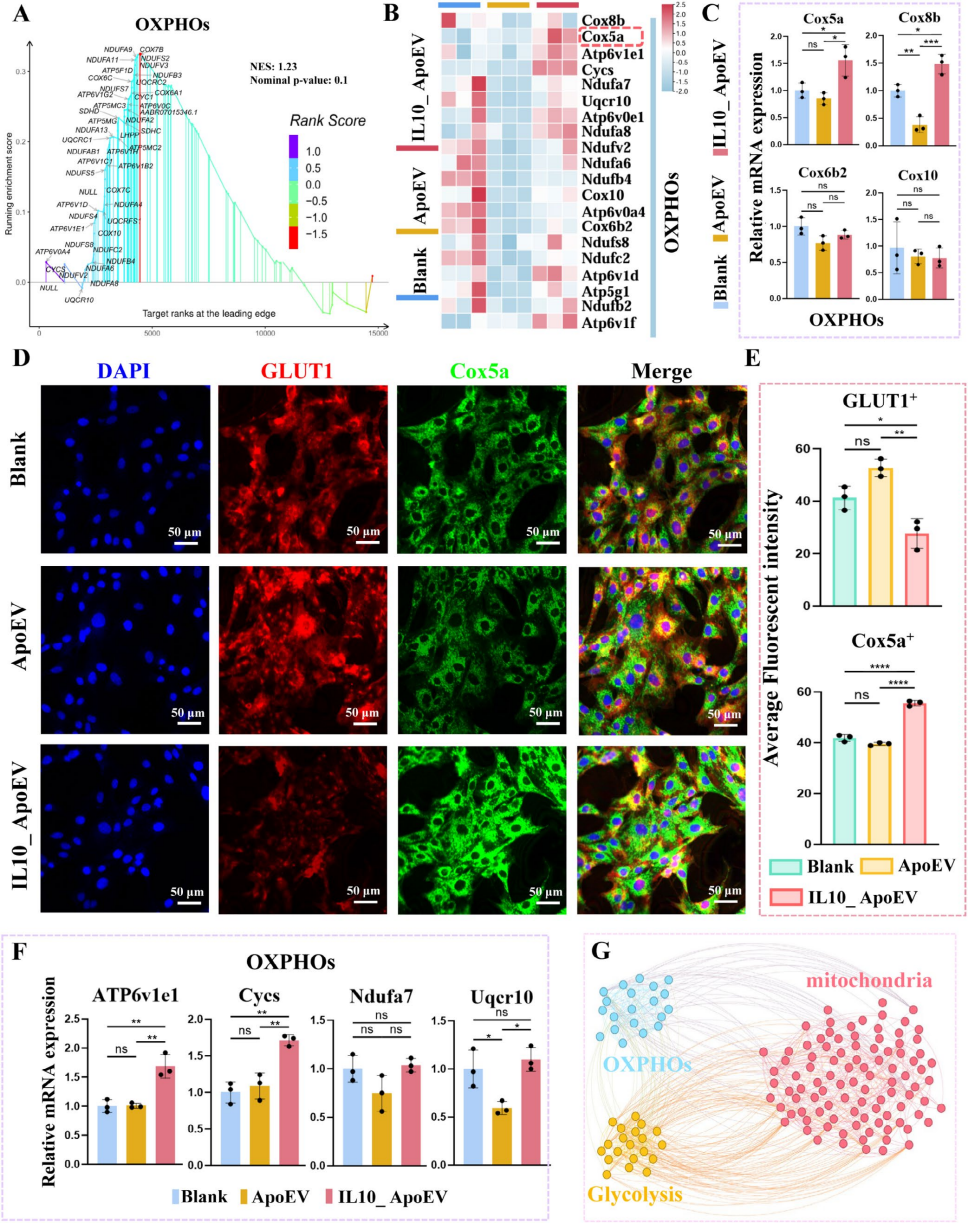
Modulation of OXPHOS and glycolytic metabolism by IL10_ApoEV in fibroblasts. (A) GSEA identifying OXPHOS as one of the affected pathways upon IL10_ApoEV treatment. (B) Heatmap representation of OXPHOS‐related gene expression, highlighting transcriptional changes across different experimental groups. (C) RT‐qPCR quantification of representative OXPHOS genes. (D) Immunofluorescence staining of fibroblasts for GLUT1 (a glycolytic marker, red) and Cox5a (an OXPHOS marker, green), with DAPI counterstaining for nuclei (blue). Merged images reveal a metabolic shift characterized by reduced GLUT1 and enhanced Cox5a expression following IL10_ApoEV treatment. (E) Semi‐quantitative fluorescence intensity analysis of GLUT1 and Cox5a, demonstrating significant downregulation of glycolysis and upregulation of OXPHOS. (F) RT‐qPCR validation of additional OXPHOS‐related genes. (G) Network correlation analysis illustrating the functional interplay between OXPHOS, glycolysis, and mitochondrial pathways.

Gene Set Enrichment Analysis (GSEA) enrichment analysis revealed that the mitochondrial OXPHOS pathway was upregulated in the IL10_ApoEV group (Figure 6A). This enrichment was further corroborated by heatmap visualization and RT‐qPCR, which highlighted the transcriptional upregulation of multiple OXPHOS‐related genes (Figure 6B,C,F). Consistently, immunofluorescence staining revealed a pronounced increase in Cox5a expression, accompanied by a reduction in GLUT1, a glycolytic marker, suggesting a metabolic reprogramming from glycolysis toward OXPHOS (Figure 6D). Semi‐quantitative fluorescence intensity analysis further validated these findings, showing significantly decreased GLUT1 and enhanced Cox5a expression levels in the IL10_ApoEV group (Figure 6E). Considering the intimate functional relationship between OXPHOS and mitochondrial activity, correlation network analysis was performed, which demonstrated strong interconnections between OXPHOS genes, glycolytic regulators, and mitochondrial pathways (Figure 6G).

**FIGURE 7:**
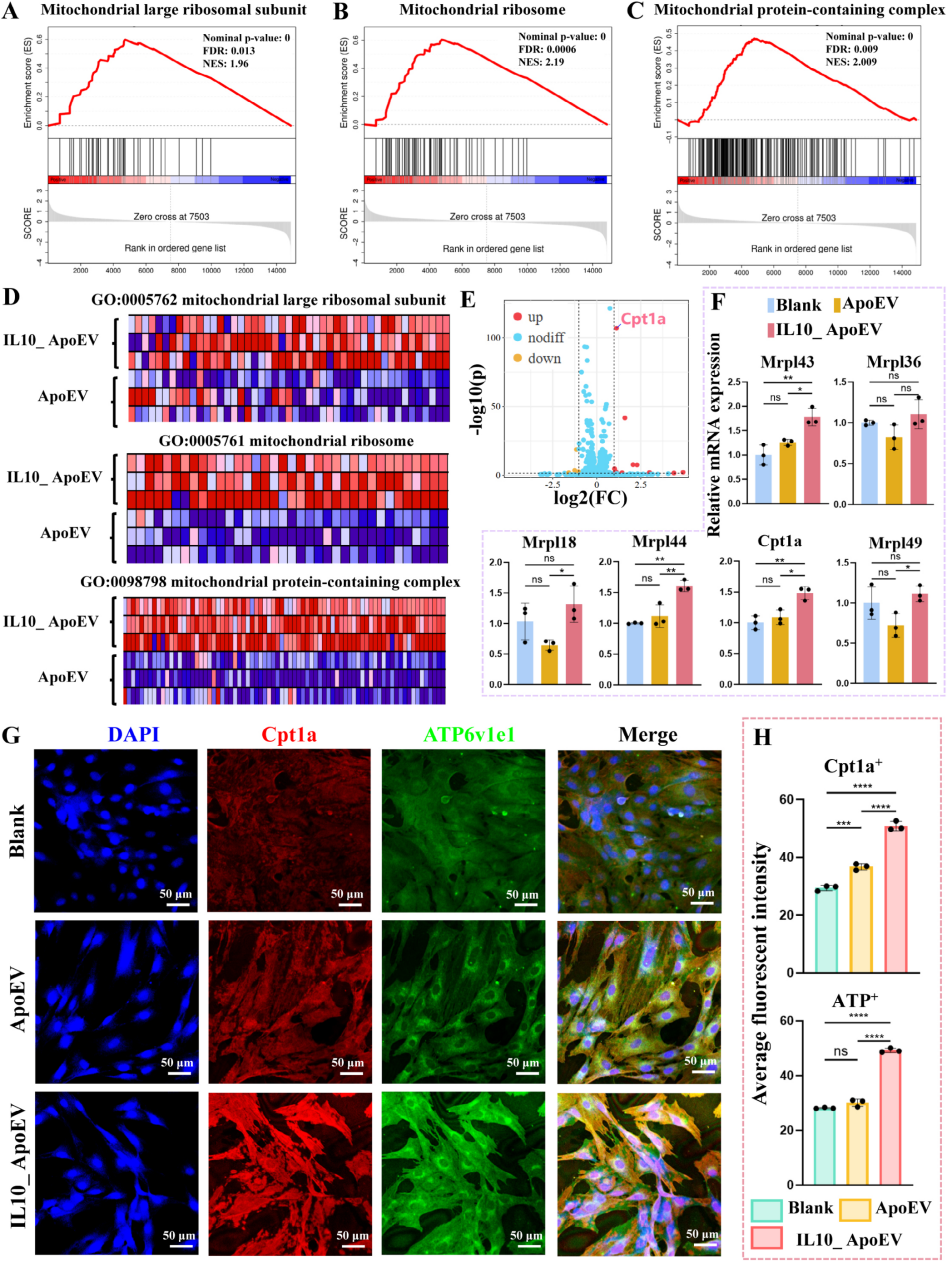
IL10_ApoEV regulates fibroblast metabolic pathways through modulation of mitochondrial function. (A–C) GSEA showing significant enrichment of mitochondrial‐related pathways, including the mitochondrial large ribosomal subunit, mitochondrial ribosome, and mitochondrial protein‐containing complex, in fibroblasts treated with IL10_ApoEV. Thresholds of |NES| > 1, NOM *p*‐value < 0.05 and FDR < 0.25 were applied to define pathway significance. (D) Heatmaps illustrating the transcriptional upregulation of genes associated with these mitochondrial pathways. (E) Volcano plot highlighting differentially expressed genes, with *Cpt1a* prominently upregulated in the IL10_ApoEV group. (F) RT‐qPCR validation of representative genes. (G) Immunofluorescence staining demonstrating increased expression and localization of mitochondrial markers *Cpt1a* (a rate‐limiting enzyme for fatty acid oxidation) and *ATP6v1e1* (a subunit of ATP synthase complex) in fibroblasts following IL10_ApoEV treatment. (H) Quantitative fluorescence intensity analysis confirming significantly elevated levels of *Cpt1a* and *ATP6v1e1* in the IL10_ApoEV group.

Further GSEA enrichment analysis demonstrated that mitochondrial‐related pathways, including the mitochondrial large ribosomal subunit, mitochondrial ribosome, and mitochondrial protein‐containing complex, were significantly upregulated in the IL10_ApoEV group, with robust statistical significance (Figure 7A–C). Consistently, heatmap visualization revealed broad transcriptional activation of genes within these pathways, while RT‐qPCR validation confirmed significant upregulation of representative genes such as *Mrpl43*, *Mrpl18*, *Mrpl44*, *Mrpl49*, and *Cpt1a* (Figure 7D–F). To directly evaluate mitochondrial functionality, immunofluorescence staining was performed for *Cpt1a*, a key enzyme regulating fatty acid transport into mitochondria, and *ATP6v1e1*, a subunit of the ATP synthase complex. Both proteins exhibited markedly enhanced expression and mitochondrial localization in fibroblasts treated with IL10_ApoEV, paralleled by increased intracellular ATP production (Figures 7G,H, S3). Collectively, these results provide strong evidence that IL10_ApoEV treatment reprograms fibroblast metabolism by promoting a metabolic shift from glycolysis toward enhanced mitochondrial OXPHOs following endocytotic uptake. This bioenergetic reconfiguration not only highlights the capacity of IL10_ApoEV to optimise energy efficiency but also suggests its potential in supporting fibroblast‐driven tissue repair and regenerative processes.

### 2.5 Effects of IL10_ApoEV on the Sonic Hedgehog Pathway and Abnormal ECM Formation

The Hedgehog signalling pathway plays a pivotal role in regulating embryogenesis, tissue homeostasis, regeneration, and pathological fibrosis [48, 49]. It operates through a canonical cascade involving Hedgehog ligands such as Sonic hedgehog (Shh), the transmembrane receptor Patched1 (Ptch1), signal transducer Smoothened (Smo), and the downstream Gli family transcription factors, primarily Gli1 [50]. Aberrant activation of this pathway has been linked to chronic inflammatory diseases, including hepatic and pulmonary fibrosis, by promoting fibroblast activation, inflammatory cytokine secretion, and excessive ECM deposition [51, 52, 53]. Moreover, while Hedgehog signalling can facilitate epidermal stem cell proliferation and wound healing, it may also drive excessive tissue remodelling and fibrotic scar formation under pathological conditions [54].

**FIGURE 8:**
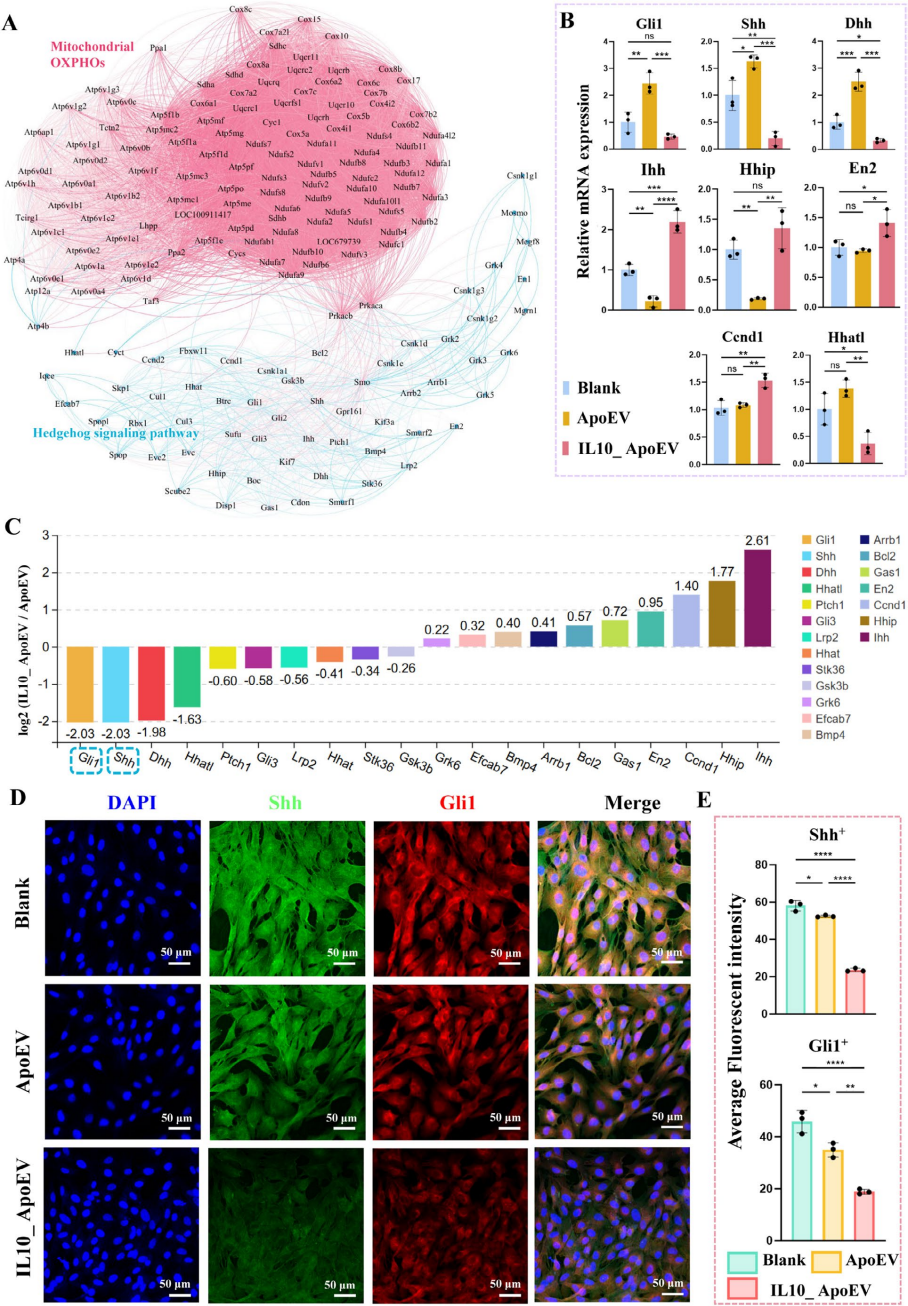
IL10_ApoEV modulates the hedgehog signalling pathway and mitochondrial OXPHOs Crosstalk. (A) Correlation network analysis illustrating the interplay between mitochondrial oxidative phosphorylation (OXPHOs) genes (pink) and Hedgehog signalling pathway genes (blue), suggesting potential regulatory interactions in fibroblasts following IL10_ApoEV treatment. (B) RT‐qPCR analysis quantifying mRNA expression levels of key Hedgehog pathway genes. (C) Bar graph depicting the log2 fold change (log2FC) of Hedgehog pathway genes between IL10_ApoEV and ApoEV groups. (D) Representative immunofluorescence images showing reduced protein expression of Shh and Gli1 (green and red, respectively) following IL10_ApoEV treatment, with DAPI staining nuclei (blue). (E) Semi‐quantitative fluorescence intensity analysis of Shh^+^ and Gli1^+^ cells.

To explore the relationship between mitochondrial metabolism and profibrotic signalling, we performed KEGG‐based gene interaction network analysis, which revealed a close association between mitochondrial OXPHOs genes and components of the Hedgehog signalling pathway in fibroblasts treated with IL10_ApoEV (Figure 8A). This correlation suggests potential crosstalk between cellular energy metabolism and fibrotic transcriptional programs. Further analysis of differentially expressed genes in the Hedgehog pathway identified Shh and Gli1 as significantly downregulated in the IL10_ApoEV group compared to ApoEV and Blank controls. This suppression was consistently validated at both the transcript level by RT‐qPCR (Figure 8B), log_2_ fold change (log2FC) analysis from RNA sequencing (Figure 8C), and at the protein level by immunofluorescence staining (Figure 8D), with Shh and Gli1 exhibiting marked reductions in expression and nuclear localization. Quantification of the fluorescence intensity further confirmed the inhibitory effect of IL10_ApoEV on Shh and Gli1 activity (Figure 8E). These findings suggest that IL10_ApoEV suppresses Hedgehog signalling, particularly Shh and Gli1 in fibroblasts, potentially through mitochondrial reprogramming, thereby contributing to an anti‐fibrotic and pro‐regenerative cellular phenotype.

To further interrogate the mechanistic link between OXPHOS activity and Hedgehog signalling inhibition, we performed a pharmacological intervention using oligomycin, a mitochondrial ATP synthase inhibitor. Notably, oligomycin treatment rescued the expression of Shh and Gli1, reversing the suppressive effect induced by IL10_ApoEV (Figure S4). This suggests that enhanced mitochondrial activity contributes directly to the downregulation of Hedgehog signalling, positioning OXPHOS as a metabolic checkpoint in modulating fibroblast profibrotic responses.

**FIGURE 9:**
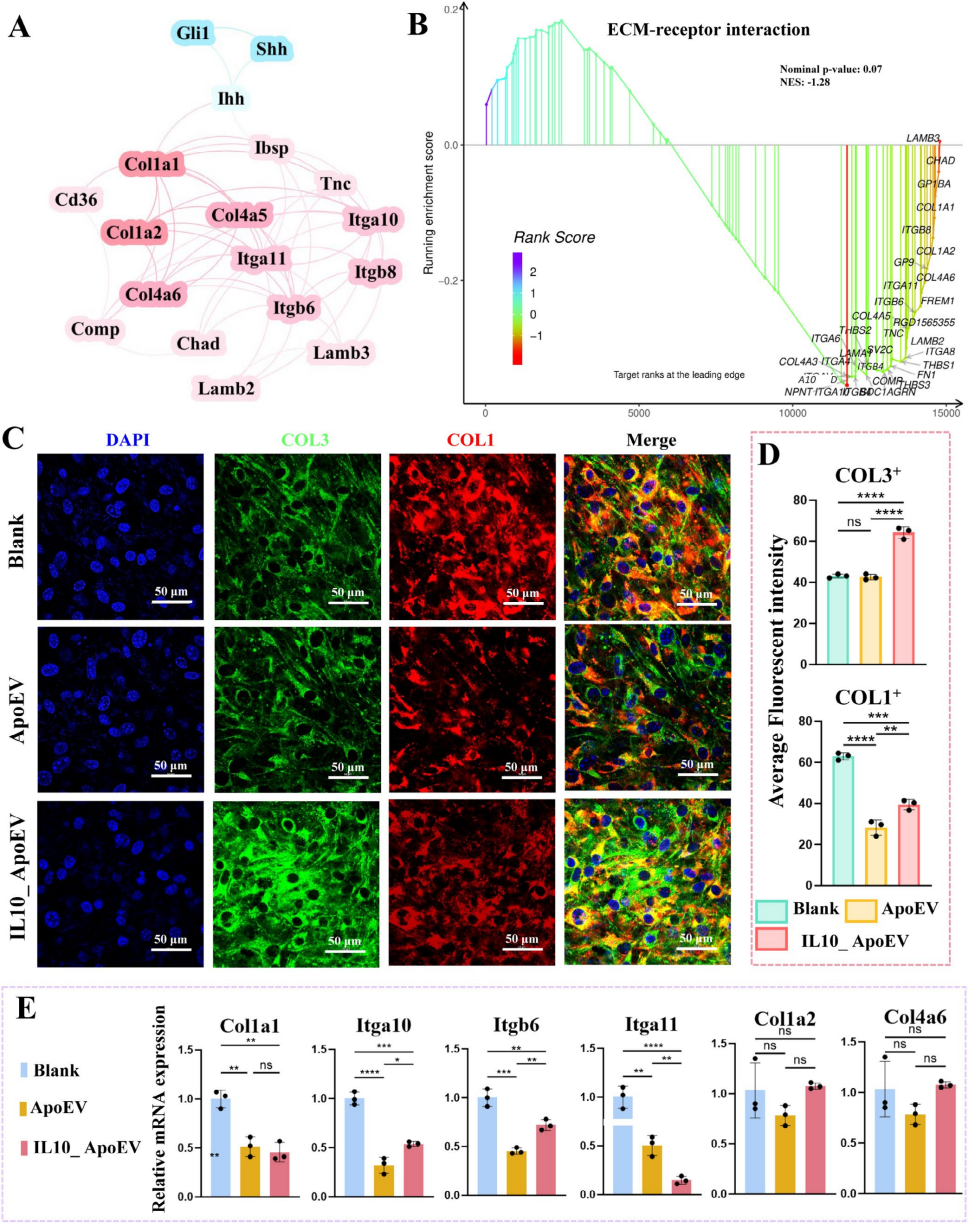
IL10_ApoEV modulates ECM deposition and ECM remodelling through the Hedgehog signalling. (A) Gene interaction network showing the correlation between Hedgehog signalling molecules (e.g., *Gli1*, *Shh*, *Ihh*) and ECM components involved in ECM‐receptor interaction. (B) GSEA of the ECM‐receptor interaction pathway. (C) Representative immunofluorescence staining for COL3 (green) and COL1 (red) across different treatment groups. (D) Semi‐quantitative analysis of fluorescence intensity for COL3^+^ and COL1^+^ regions. (E) RT‐qPCR results.

To further elucidate the downstream effects of Gli1 and Shh signalling suppression, we investigated its interplay with ECM remodelling. Gene interaction network analysis revealed correlations between Hedgehog signalling mediators (Gli1, Shh, Ihh) and key ECM‐receptor components (Figure 9A). These associations suggest a regulatory axis through which Hedgehog activity may influence fibrotic matrix deposition. GSEA of the ECM‐receptor interaction pathway indicated downregulation in the IL10_ApoEV group, implying reduced ECM activation and a potential alleviation of fibrotic remodelling (Figure 9B). Immunofluorescence staining further demonstrated that IL10_ApoEV‐treated fibroblasts exhibited a pronounced increase in type III collagen (COL3) expression, while type I collagen (COL1, a hallmark of stiff, fibrotic tissue) was notably reduced (Figure 9C). Quantitative fluorescence analysis confirmed this shift, with COL3^+^ area significantly increased and COL1^+^ area markedly decreased in the IL10_ApoEV group compared to ApoEV and Blank controls (Figure 9D). These observations were corroborated by RT‐qPCR results, which showed downregulation of most key ECM receptor‐related genes (Figure 9E). Together, these findings underscore the role of IL10_ApoEV in modulating the Hedgehog–ECM receptor interaction axis to attenuate fibrotic ECM accumulation and promote a regenerative collagen profile. By reducing COL1 and enhancing COL3 expression, IL10_ApoEV fosters a microenvironment more conducive to scarless healing, soft tissue elasticity, and functional tissue repair, which offers a promising strategy for mitigating pathological fibrosis following tissue injury.

Skin wound healing is a precisely orchestrated process involving immune cell recruitment, cytokine signaling, apoptosis, and ECM remodeling. ApoEVs, released from dying cells, actively participate in this microenvironment by modulating macrophage phagocytosis and delivering signaling molecules. IL‐10, a key anti‐inflammatory cytokine, mitigates scar formation by suppressing pro‐inflammatory mediators, ECM overproduction, and myofibroblast transdifferentiation. In addition, we confirmed that IL10‐ApoEVs retained the anti‐inflammatory function of IL‐10 by reducing M1 polarization (iNOS^+^) and enhancing M2 polarization (CD206^+^) in macrophages within the wound site, as shown by immunofluorescence and semi‐quantitative analysis after 3 days of collagen scaffold implantation (Figure S5).

## 3 Conclusion

In summary, our study reveals that IL10‐ApoEVs profoundly modulate fibroblast metabolism by enhancing mitochondrial oxidative phosphorylation (OXPHOs) while suppressing glycolysis, thereby driving a metabolic shift that favors tissue regeneration over fibrosis. This reprogramming is accompanied by significant downregulation of the Hedgehog signaling pathway, notably Shh and Gli1, which are key drivers of fibrotic ECM deposition. IL10‐ApoEV treatment promotes the preferential synthesis of type III collagen (associated with more elastic, functional tissue) while reducing type I collagen accumulation, a hallmark of hypertrophic scarring. These effects collectively suggest that IL10‐ApoEVs not only influence cellular energy metabolism but also exert broader regulatory control over fibroblast behavior, collagen remodeling, and scar formation. The therapeutic potential of IL10‐ApoEVs thus lies in their ability to coordinate immunomodulation, metabolic tuning, and fibrotic suppression to promote scarless wound healing. However, limitations in ApoEV yield due to low production and ultracentrifugation‐based purification remain a technical hurdle. Future efforts will focus on optimizing isolation techniques to improve both the quantity and purity of IL10‐ApoEVs, facilitating their translational application in regenerative medicine and wound repair.

## 4 Materials and Methods

### 4.1 Preparation and Characterization of IL‐10 Treating Apoptotic Vesicles

The skin layer was harvested from the dorsal region of 4‐week‐old Sprague–Dawley (SD) rats, and fibroblasts were isolated and subsequently stimulated with IL‐10 for 24 h. IL‐10‐stimulated fibroblasts and untreated fibroblasts were treated with staurosporine (Cell Signalling Technology) at 500 nM for 16 h to induce apoptosis, based on the support from existing literature [9, 55]. Then, the cells and culture medium were collected and centrifuged at 800 g for 10 min to remove cells and debris. The supernatant was transferred to a new tube and centrifuged at 16,000 g for 30 min to concentrate ApoEV. The supernatant was removed and the precipitate was suspended in PBS for subsequent experiments. To validate the identity and properties of the isolated ApoEVs: Morphology was assessed using TEM, confirming the irregular, heterogeneous structure characteristic of ApoEVs. The size distribution of ApoEVs was measured using nanoparticle tracking analysis (NTA) with the NanoSight NS300 system (NanoSight Technology, UK), providing detailed insights into the dimensional profile of the vesicle population. Protein expression was analysed via Western blotting and ELISA kits, quantifying the presence of apoptosis‐specific proteins. Additionally, SEM was used to visualise the interaction between ApoEVs and collagen scaffolds, clarifying their adherence and structural alignment on the scaffold surface. Through the integration of these criteria and experimental methods, alongside references to established literature [9], we accurately confirmed that the studied vesicles are apoptotic vesicles. This rigorous validation ensures the accuracy and reliability of subsequent research involving these vesicles.

### 4.2 Protein Isolation, Western Blot Analysis and ELISA Assay

Protein samples were extracted using RIPA buffer (Cell Signalling Technology) supplemented with a protease inhibitor cocktail (Cell Signalling Technology). The total protein concentration was determined using the BCA protein assay kit. Equal amounts of protein were resolved by SDS‐polyacrylamide gel electrophoresis and then transferred onto polyvinylidene fluoride membranes. The membranes were blocked with 5% bovine serum albumin (BSA) in Tris‐buffered saline containing 0.1% Tween 20 (TBST) at room temperature for 2 h, followed by incubation with primary antibodies overnight at 4°C. After three washes with TBST, the membranes were incubated with the corresponding secondary antibodies at room temperature for 2 h. Protein bands were detected using the Western‐Light chemiluminescence detection system (Tanon), and the digital images were quantitatively analysed using ImageJ software. The primary antibodies used included β‐actin, Histone H3, and Cleaved Caspase‐3 (all from Cell Signalling Technology). The concentrations of IL‐10 were measured using ELISA kits according to the manufacturer's instructions.

### 4.3 Integration of Collagen Scaffold With ApoEV and IL10‐ApoEV

Tropocollagen solution (#354236, Corning, USA) was diluted to a concentration of 0.1 mg/mL with 0.1 M NaOH in sterile PBS (pH = 9.2). A volume of 200 μL of the collagen solution was added to each well of a 24‐well plate to form appropriately sized collagen scaffolds at 37°C and 100% humidity for 24 h. Subsequently, the collagen scaffolds were crosslinked using 100 μL of a sterile solution containing 0.3 M 1‐ethyl‐3‐(3‐dimethylaminopropyl)‐carbodiimide (EDC) and 0.06 M N‐hydroxysulfo‐succinimide sodium salt (NHS) for 4 h at 37°C. The resulting gelatinous collagen scaffolds had an approximate thickness of 3 mm and a diameter of 10 mm. In the animal experiments, ApoEV and IL10_ApoEV were individually combined with collagen scaffolds and subsequently implanted into the skin defects on the rat dorsal region.

### 4.4 Animal Surgery and Sample Preparation

Male Sprague–Dawley rats from the Experimental Animal Center of Nanjing University (SYXK (Jiangsu) 2021‐0034) were used as the experimental animals. All experiments involving animal use were approved by the IACUC of Nanjing University (Approval No: IACUC‐D2403184). Animal surgery and sample preparation were performed under general anaesthesia (Zoletil 50, 0.2 mL/100 g). Following general anaesthesia, the dorsal region of the rats was shaved and disinfected using iodophor. Full‐thickness skin defects were surgically induced on the dorsum. Collagen implants were subsequently introduced into the defects and secured with sterile bandages. Wound status was documented on days 0, 3, 7, and 15 post‐surgery. The animals were euthanized on days 3 and 15 post‐implantation for histological analysis.

### 4.5 Histological Analysis

The tissue samples were fixed in a 4% paraformaldehyde solution buffered with 0.1 M phosphate buffer for 24 h, followed by gradient dehydration and embedding. For histological staining, the samples were processed using Mayer's haematoxylin (Servicebio, China) and eosin (Servicebio, China), Masson trichrome staining kit (Beyotime, China), PGP9.5 antibody (1:100; Servicebio), and goat anti‐mouse/rabbit secondary antibody (Genentech). Imaging was performed using the Aperio AT2 system (Leica Biosystems), and semi‐quantitative analysis was conducted using ImageJ software (version 1.46) and ImageScope software.

### 4.6 qRT‐PCR Immunofluorescence Staining and ATP Detection

The total RNA was quantified using a Nanodrop spectrophotometer (Thermo Fisher, USA) and subsequently reverse‐transcribed into complementary DNA (cDNA) using the PrimeScript RT Master Mix (Takara, Japan). Equivalent amounts of cDNA were subjected to RT‐qPCR analysis with the Hieff qPCR SYBR Green Master Mix (Yeasen, China) on an ABI two‐step system (Applied Biosystems, USA). The primer sequences are provided in the Data S1 (Table S1). Relative gene expression levels were calculated using the 2^−ΔΔCt^ method, normalized to GAPDH.

The primary antibodies of immunofluorescence staining used were as follows: CD31 (1:100, Affbiotech), α‐SMA (1:100, Affbiotech), TGF‐β (1:100, Servicebio), Actin (1:100, Servicebio), PKH26 (1:100, Sigma), GLUT1 (1:100, Servicebio), Cox5a (1:100, Servicebio), Cpt1a (1:100, Proteintech), ATP6v1e1 (1:100, Servicebio), Shh (1:100, Proteintech), Gli1 (1:100, Proteintech), Col1 (1:100, GB124197), and Col3 (1:100, GB111629). Secondary antibodies included Alexa Fluor 594 goat anti‐mouse (Emarbio, China) and Alexa Fluor 647 goat anti‐rabbit (Beyotime, China). The concentration of the mitochondrial inhibitor oligomycin (Abcam, China) used was 50 ng/mL, and the treatment duration was 5 min.

Luciferin was catalyzed by firefly luciferase to produce chemiluminescent oxyluciferin. ATP provided energy for this reaction. The chemiluminescence intensity was proportional to the ATP concentration. ATP production detection was performed according to the manufacturer's protocol. Briefly, the standard ATP stock solution was diluted into aliquots with a series of concentrations, including 0.01, 0.025, 0.1, 0.25, 1.0, and 2.5 μM. Each cell‐laden hydrogel was immersed in 200 μL of lysis buffer. Then, the sample was centrifuged at 12000 g for 10 min at 4°C. The obtained supernatant was stored in a 1.5 mL Eppendorf tube in an ice bath. Next, 100 μL of ATP detection buffer was added to each well to consume the background ATP. Finally, 20 μL of standard solution or sample was added into each well, and the absorbance was measured by a chemiluminescence detector in a microplate reader.

### 4.7 RNA Sequencing and Omics Analysis

Total RNA was extracted from fibroblasts that were respectively stimulated with apoEV, IL10_apoEV and without any treatment, using TRIzol reagent (Invitrogen) homogenization (30 Hz, 2 min). Following chloroform phase separation, RNA was precipitated with isopropanol (−20°C overnight), washed with 75% DEPC‐ethanol, and resuspended in 30 μL DEPC‐water. Quality was verified via NanoDrop 2000 (260/280: 1.8–2.1) and Agilent 2100 Bioanalyzer (RIN > 8.0). cDNA libraries were constructed from 1 μg RNA using NEBNext Ultra Library Prep Kit (NEB): mRNA enrichment with oligo(dT) beads, ~200 bp fragmentation, strand synthesis, end repair, adaptor ligation, and 12‐cycle PCR amplification. Libraries (250–300 bp) were validated by Bioanalyzer and qPCR. Sequencing was performed on Illumina NovaSeq 6000 (BGI) with PE150 reads, generating ~6 Gb/sample. Raw data were filtered via Trimmomatic (v0.39; Phred ≥ 20, < 10% N, adapter removal) after FastQC (v0.11.9) assessment. Reads were mapped to GRCh38 using HISAT2 (v2.2.1), with counts generated via featureCounts (v2.0.3; Gencode v38). DEGs (adjusted *p* < 0.05, |log2FC| > 1) were identified using DESeq2 (v1.34.0) in R (v4.1.2). Functional enrichment (GO/KEGG) was done via clusterProfiler (v4.2.2; adjusted *p* < 0.05). PPI networks (STRING v11.5), heatmaps, volcano plot and GSEA were visualized with Gephi (v0.9.2), TBtools (v1.09876), GraphPad Prism (v9.3.1), and OmicShare tools.

### 4.8 Statistical Analysis

All experiments were repeated at least three times independently. The representative SEM, immunofluorescence, and histological section staining images were presented. All the experimental results were expressed as means ± standard deviations (s.d.). One‐way (for one independent variable) analysis of variance (ANOVA) followed by Tukey's multiple comparison post hoc test was conducted unless otherwise stated. Statistical analysis of data was performed by GraphPad Prism (v 8.0.1). In all cases, *p*‐value below 0.05 was considered statistically significant (*p < 0.05; **p < 0.01; ***p < 0.001; ****p < 0.0001).

## Author Contributions

**Yang Zou:** writing – original draft, investigation, formal analysis. **Jinglun Zhang:** investigation, conceptualization, writing. **Wenxuan Mao, Shuting Jiang, Cheng Xu and Jiayi Meng:** investigation. **Heng Dong:** writing – review and editing. **Yongbin Mou:** writing – review and editing.

## Conflicts of Interest

The authors declare no conflicts of interest.

## Supporting information


**Data S1:** Supporting Information.

## Data Availability

The data that support the findings of this study are available from the corresponding author upon reasonable request.

## References

[1] G. C. Gurtner , S. Werner , Y. Barrandon , and M. T. Longaker , “Wound Repair and Regeneration,” Nature 453, no. 7193 (2008): 314–321. 18480812 10.1038/nature07039

[2] X. Cao , X. Wu , Y. Zhang , X. Qian , W. Sun , and Y. Zhao , “Emerging Biomedical Technologies for Scarless Wound Healing,” Bioactive Materials 42 (2024): 449–477. 39308549 10.1016/j.bioactmat.2024.09.001 PMC11415838

[3] Y. Z. Lu , B. Nayer , S. K. Singh , et al., “CGRP Sensory Neurons Promote Tissue Healing via Neutrophils and Macrophages,” Nature 628, no. 8008 (2024): 604–611. 38538784 10.1038/s41586-024-07237-y PMC11023938

[4] H. Nagano , N. Mizuno , H. Sato , et al., “Skin Graft With Dermis and Appendages Generated in Vivo by Cell Competition,” Nature Communications 15, no. 1 (2024): 3366. 10.1038/s41467-024-47527-7 PMC11058811 38684678

[5] M. Jarvis , U. Schulz , A. Dickinson , et al., “The Detection of Apoptosis in a Human in Vitro Skin Explant Assay for Graft Versus Host Reactions,” Journal of Clinical Pathology 55 (2002): 127–132. 11865008 10.1136/jcp.55.2.127 PMC1769581

[6] S. Mascharak , H. E. Desjardins‐Park , M. F. Davitt , et al., “Preventing Engrailed‐1 Activation in Fibroblasts Yields Wound Regeneration Without Scarring,” Science 372, no. 6540 (2021): eaba2374. 33888614 10.1126/science.aba2374 PMC9008875

[7] B. A. Shook , R. R. Wasko , G. C. Rivera‐Gonzalez , et al., “Myofibroblast Proliferation and Heterogeneity Are Supported by Macrophages During Skin Repair,” Science 362, no. 6417 (2018): eaar2971. 30467144 10.1126/science.aar2971 PMC6684198

[8] J. Lee , C. C. Rabbani , H. Gao , et al., “Hair‐Bearing Human Skin Generated Entirely From Pluripotent Stem Cells,” Nature 582, no. 7812 (2020): 399–404. 32494013 10.1038/s41586-020-2352-3 PMC7593871

[9] L. Ma , C. Chen , D. Liu , et al., “Apoptotic Extracellular Vesicles Are Metabolized Regulators Nurturing the Skin and Hair,” Bioactive Materials 19 (2023): 626–641. 35600968 10.1016/j.bioactmat.2022.04.022 PMC9109130

[10] M. Hosseini and A. Shafiee , “Engineering Bioactive Scaffolds for Skin Regeneration,” Small 17, no. 41 (2021): 2101384. 10.1002/smll.202101384 34313003

[11] C. Liang , V. Dudko , O. Khoruzhenko , et al., “Stiff and Self‐Healing Hydrogels by Polymer Entanglements in Co‐Planar Nanoconfinement,” Nature Materials 24, no. 4 (2025): 599–606. 40055539 10.1038/s41563-025-02146-5 PMC11961364

[12] M. Kurita , T. Araoka , T. Hishida , et al., “In Vivo Reprogramming of Wound‐Resident Cells Generates Skin Epithelial Tissue,” Nature 561, no. 7722 (2018): 243–247. 30185909 10.1038/s41586-018-0477-4 PMC9651909

[13] S. Mascharak , M. Griffin , H. E. Talbott , et al., “Inhibiting mechanotransduction prevents scarring and yields regeneration in a large animal model,” Science Translational Medicine 17, no. 786 (2025): eadt6387. 39970235 10.1126/scitranslmed.adt6387 PMC12893899

[14] K. Lecomte , A. Toniolo , and E. Hoste , “Cell Death as an Architect of Adult Skin Stem Cell Niches,” Cell Death and Differentiation 31, no. 8 (2024): 957–969. 38649745 10.1038/s41418-024-01297-3 PMC11303411

[15] Y. Qu , Y. He , B. Meng , et al., “Apoptotic Vesicles Inherit SOX2 From Pluripotent Stem Cells to Accelerate Wound Healing by Energizing Mesenchymal Stem Cells,” Acta Biomaterialia 149 (2022): 258–272. 35830925 10.1016/j.actbio.2022.07.009

[16] M. Yusupova , R. Ankawa , Y. Yosefzon , D. Meiri , I. Bachelet , and Y. Fuchs , “Apoptotic Dysregulation Mediates Stem Cell Competition and Tissue Regeneration,” Nature Communications 14, no. 1 (2023): 7547. 10.1038/s41467-023-41684-x PMC10662150 37985759

[17] G. Tian , H. Yin , J. Zheng , et al., “Promotion of Osteochondral Repair Through Immune Microenvironment Regulation and Activation of Endogenous Chondrogenesis via the Release of Apoptotic Vesicles From Donor MSCs,” Bioactive Materials 41 (2024): 455–470. 39188379 10.1016/j.bioactmat.2024.07.034 PMC11347043

[18] L. Chen , L. Cheng , Z. Wang , et al., “Conditioned Medium‐Electrospun Fiber Biomaterials for Skin Regeneration,” Bioactive Materials 6, no. 2 (2021): 361–374. 32954054 10.1016/j.bioactmat.2020.08.022 PMC7481508

[19] L. Jiang , J. Dong , M. Jiang , et al., “3D‐Printed Multifunctional Bilayer Scaffold With Sustained Release of Apoptotic Extracellular Vesicles and Antibacterial Coacervates for Enhanced Wound Healing,” Biomaterials 318 (2025): 123196. 39965422 10.1016/j.biomaterials.2025.123196

[20] M. Zhao , M. Kang , J. Wang , et al., “Stem Cell‐Derived Nanovesicles Embedded in Dual‐Layered Hydrogel for Programmed ROS Regulation and Comprehensive Tissue Regeneration in Burn Wound Healing,” Advanced Materials 36, no. 32 (2024): 2401369. 10.1002/adma.202401369 38822749

[21] F. Li , Q. Huang , J. Chen , et al., “Apoptotic Cells Activate the “Phoenix Rising” Pathway to Promote Wound Healing and Tissue Regeneration,” Science Signaling 3, no. 110 (2010): ra13‐ra13. 20179271 10.1126/scisignal.2000634 PMC2905599

[22] Y. Fuchs , “The Therapeutic Promise of Apoptosis,” Science 363, no. 6431 (2019): 1050–1051. 30846591 10.1126/science.aaw3607

[23] I. Liebold , A. Al Jawazneh , C. Casar , et al., “Apoptotic Cell Identity Induces Distinct Functional Responses to IL‐4 in Efferocytic Macrophages[J],” Science 384, no. 6691 (2024): eabo7027. 38574142 10.1126/science.abo7027

[24] B. Zhao , M. Wei , X. Zhou , et al., “Supramolecular Assembly‐Enabled Transdermal Therapy of Hypertrophic Scarring Through Concurrent Ferroptosis‐Apoptosis,” Advanced Functional Materials 35, no. 9 (2025): 2416011.

[25] S. Qian , J. Mao , Q. Zhao , et al., ““Find‐Eat” Strategy Targeting Endothelial Cells via Receptor Functionalized Apoptotic Body Nanovesicle,” Science Bulletin 68, no. 8 (2023): 826–837. 36973107 10.1016/j.scib.2023.03.030

[26] Y. Fuchs and H. Steller , “Programmed Cell Death in Animal Development and Disease,” Cell 147, no. 4 (2011): 742–758. 22078876 10.1016/j.cell.2011.10.033 PMC4511103

[27] M. P. Baar , R. M. Brandt , D. A. Putavet , et al., “Targeted apoptosis of senescent cells restores tissue homeostasis in response to chemotoxicity and aging,” Cell 169, no. 1 (2017): e116. 10.1016/j.cell.2017.02.031 PMC5556182 28340339

[28] X. Li , S. Li , X. Fu , and Y. Wang , “Apoptotic Extracellular Vesicles Restore Homeostasis of the Articular Microenvironment for the Treatment of Rheumatoid Arthritis,” Bioactive Materials 35 (2024): 564–576. 38469201 10.1016/j.bioactmat.2023.11.019 PMC10925912

[29] Y. Zhou , L. Bao , S. Gong , et al., “T Cell‐Derived Apoptotic Extracellular Vesicles Hydrolyze cGAMP to Alleviate Radiation Enteritis via Surface Enzyme ENPP1,” Advanced Science 11, no. 31 (2024): 2401634. 38888507 10.1002/advs.202401634 PMC11336903

[30] Y. Chen , X. Ning , S. Zhu , et al., “Phospholipid‐Modified Titanium Surface‐Loaded Apoptotic Extracellular Vesicles Promote Early Angiogenesis and Improve Implant Osseointegration Through Immune Regulation,” Advanced Materials Interfaces 11, no. 24 (2024): 2400146.

[31] Y. Wang , C. Liu , J. Pang , et al., “The Extra‐Tumoral Vaccine Effects of Apoptotic Bodies in the Advancement of Cancer Treatment,” Small 21, no. 9 (2025): 2410503. 39871756 10.1002/smll.202410503 PMC11878267

[32] A. Liu , P. Peng , C. Wei , et al., “Apoptotic Vesicles Derived From Mesenchymal Stem Cells Ameliorate Hypersensitivity Responses via Inducing CD8^+^ T Cells Apoptosis With Calcium Overload and Mitochondrial Dysfunction,” Advanced Science 12, no. 22 (2025): 2407446. 40089865 10.1002/advs.202407446 PMC12165088

[33] R. Wang , M. Hao , X. Kou , et al., “Apoptotic Vesicles Ameliorate Lupus and Arthritis via Phosphatidylserine‐Mediated Modulation of T Cell Receptor Signaling,” Bioactive Materials 25 (2023): 472–484. 37056273 10.1016/j.bioactmat.2022.07.026 PMC10087106

[34] M. Li , L. Liao , and W. Tian , “Extracellular Vesicles Derived From Apoptotic Cells: An Essential Link Between Death and Regeneration,” Frontiers in Cell and Developmental Biology 8 (2020): 573511. 33134295 10.3389/fcell.2020.573511 PMC7561711

[35] C. Hu , C. Chu , L. Liu , et al., “Dissecting the Microenvironment Around Biosynthetic Scaffolds in Murine Skin Wound Healing[J],” Science Advances 7, no. 22 (2021): eabf0787. 34039601 10.1126/sciadv.abf0787 PMC8153724

[36] D. J. Li , C. E. Berry , D. C. Wan , et al., “Clinical, Mechanistic, and Therapeutic Landscape of Cutaneous Fibrosis,” Science Translational Medicine 16, no. 766 (2024): eadn7871. 39321265 10.1126/scitranslmed.adn7871 PMC12093819

[37] L. E. Tracy , R. A. Minasian , and E. J. Caterson , “Extracellular Matrix and Dermal Fibroblast Function in the Healing Wound,” Advances in Wound Care 5, no. 3 (2016): 119–136. 26989578 10.1089/wound.2014.0561 PMC4779293

[38] Y. Ma , Z. Liu , L. Miao , et al., “Mechanisms Underlying Pathological Scarring by Fibroblasts During Wound Healing,” International Wound Journal 20, no. 6 (2023): 2190–2206. 36726192 10.1111/iwj.14097 PMC10333014

[39] T.‐S. Lee and L.‐Y. Chau , “Heme Oxygenase‐1 Mediates the Anti‐Inflammatory Effect of Interleukin‐10 in Mice,” Nature Medicine 8, no. 3 (2002): 240–246. 10.1038/nm0302-240 11875494

[40] R. A. Saxton , N. Tsutsumi , L. L. Su , et al., “Structure‐Based Decoupling of the Pro‐ and Anti‐Inflammatory Functions of Interleukin‐10,” Science 371, no. 6535 (2021): eabc8433. 33737461 10.1126/science.abc8433 PMC9132103

[41] W. Ouyang and A. O'garra , “IL‐10 Family Cytokines IL‐10 and IL‐22: From Basic Science to Clinical Translation,” Immunity 50, no. 4 (2019): 871–891. 30995504 10.1016/j.immuni.2019.03.020

[42] W. K. E. Ip , N. Hoshi , D. S. Shouval , S. Snapper , and R. Medzhitov , “Anti‐Inflammatory Effect of IL‐10 Mediated by Metabolic Reprogramming of Macrophages,” Science 356, no. 6337 (2017): 513–519. 28473584 10.1126/science.aal3535 PMC6260791

[43] T. Takiishi , C. E. Tadokoro , L. V. Rizzo , and L. V. de Moraes , “Early IL‐10 Production Is Essential for Syngeneic Graft Acceptance,” Journal of Leukocyte Biology 92, no. 2 (2012): 259–264. 22416256 10.1189/jlb.1111569

[44] B. Uricoli , L. A. Birnbaum , P. Do , et al., “Engineered Cytokines for Cancer and Autoimmune Disease Immunotherapy,” Advanced Healthcare Materials 10, no. 15 (2021): 2002214. 10.1002/adhm.202002214 PMC8651077 33690997

[45] X. Wang , N. K. Brown , B. Wang , et al., “Local Immunomodulatory Strategies to Prevent Allo‐Rejection in Transplantation of Insulin‐Producing Cells,” Advanced Science 8, no. 17 (2021): 2003708. 34258870 10.1002/advs.202003708 PMC8425879

[46] X.‐M. Meng , D. J. Nikolic‐Paterson , and H. Y. Lan , “TGF‐β: the Master Regulator of Fibrosis,” Nature Reviews Nephrology 12, no. 6 (2016): 325–338. 27108839 10.1038/nrneph.2016.48

[47] S. Sinha , H. D. Sparks , E. Labit , et al., “Fibroblast Inflammatory Priming Determines Regenerative Versus Fibrotic Skin Repair in Reindeer,” Cell 185, no. 25 (2022): e4725. 10.1016/j.cell.2022.11.004 PMC9888357 36493752

[48] W. Zhang , J. Lu , L. Feng , et al., “Sonic Hedgehog‐Heat Shock Protein 90β Axis Promotes the Development of Nonalcoholic Steatohepatitis in Mice,” Nature Communications 15, no. 1 (2024): 1280. 10.1038/s41467-024-45520-8 PMC10859387 38342927

[49] L.‐H. Zeng , C. Tang , M. Yao , et al., “Phosphorylation of Human Glioma‐Associated Oncogene 1 on Ser937 Regulates Sonic Hedgehog Signaling in Medulloblastoma,” Nature Communications 15, no. 1 (2024): 987. 10.1038/s41467-024-45315-x PMC10837140 38307877

[50] G. Cong , X. Zhu , X. R. Chen , et al., “Mechanisms and Therapeutic Potential of the Hedgehog Signaling Pathway in Cancer,” Cell Death Discovery 11, no. 1 (2025): 40. 39900571 10.1038/s41420-025-02327-w PMC11791101

[51] J. H. W. Distler , A.‐H. Györfi , M. Ramanujam , M. L. Whitfield , M. Königshoff , and R. Lafyatis , “Shared and Distinct Mechanisms of Fibrosis,” Nature Reviews Rheumatology 15, no. 12 (2019): 705–730. 31712723 10.1038/s41584-019-0322-7

[52] T. E. King , A. Pardo , and M. Selman , “Idiopathic Pulmonary Fibrosis,” Lancet 378, no. 9807 (2011): 1949–1961. 21719092 10.1016/S0140-6736(11)60052-4

[53] A. Omenetti , S. Choi , G. Michelotti , and A. M. Diehl , “Hedgehog Signaling in the Liver,” Journal of Hepatology 54, no. 2 (2011): 366–373. 21093090 10.1016/j.jhep.2010.10.003 PMC3053023

[54] C. H. Lim , Q. Sun , K. Ratti , et al., “Hedgehog Stimulates Hair Follicle Neogenesis by Creating Inductive Dermis During Murine Skin Wound Healing,” Nature Communications 9, no. 1 (2018): 4903. 10.1038/s41467-018-07142-9 PMC6249328 30464171

[55] G. Yu , Y. Chen , N. Yang , et al., “Apoptotic Bodies Derived From Fibroblast‐Like Cells in Subcutaneous Connective Tissue Inhibit Ferroptosis in Ischaemic Flaps via the miR‐339‐5p/KEAP1/Nrf2 Axis,” Advanced Science 11, no. 24 (2024): 2307238. 38639443 10.1002/advs.202307238 PMC11200024

