# Supplementary material for: Interleukin‐10 Derived Apoptotic Vesicles Enhance Scarless Skin Healing by Modulating Fibroblast Metabolism and Fibrosis Pathways
Source: Cell Prolif. 2025 Oct 12;59(5):e70135. doi: 10.1111/cpr.70135 (PMC13114772; doi:10.1111/cpr.70135)
Supplement: Supplementary file 1 — Data S1: Supporting Information. [file CPR-59-e70135-s001.docx]

**Supplementary file**

**Interleukin-10 Derived Apoptotic Vesicles Enhance Scarless Skin Healing by Modulating Fibroblast Metabolism and Fibrosis Pathways**

Yang Zou ^a,1^, Jinglun Zhang ^a,1^, Wenxuan Mao ^a^, Shuting Jiang ^a^, Cheng Xu ^a^, Jiayi Meng ^a^, Heng Dong ^a,*^, Yongbin Mou ^a,*^

^a^ Nanjing Stomatological Hospital, Affiliated Hospital of Medical School, Institute of Stomatology, Nanjing University, Nanjing, China.

^1^These authors as co-first author contributed equally to this work.

*Corresponding author:

[dongheng90@smail.nju.edu.cn](mailto:dongheng90@smail.nju.edu.cn) (H. Dong), [yongbinmou@nju.edu.cn](mailto:yongbinmou@nju.edu.cn) (Y. Mou).

**Supplementary Figure**


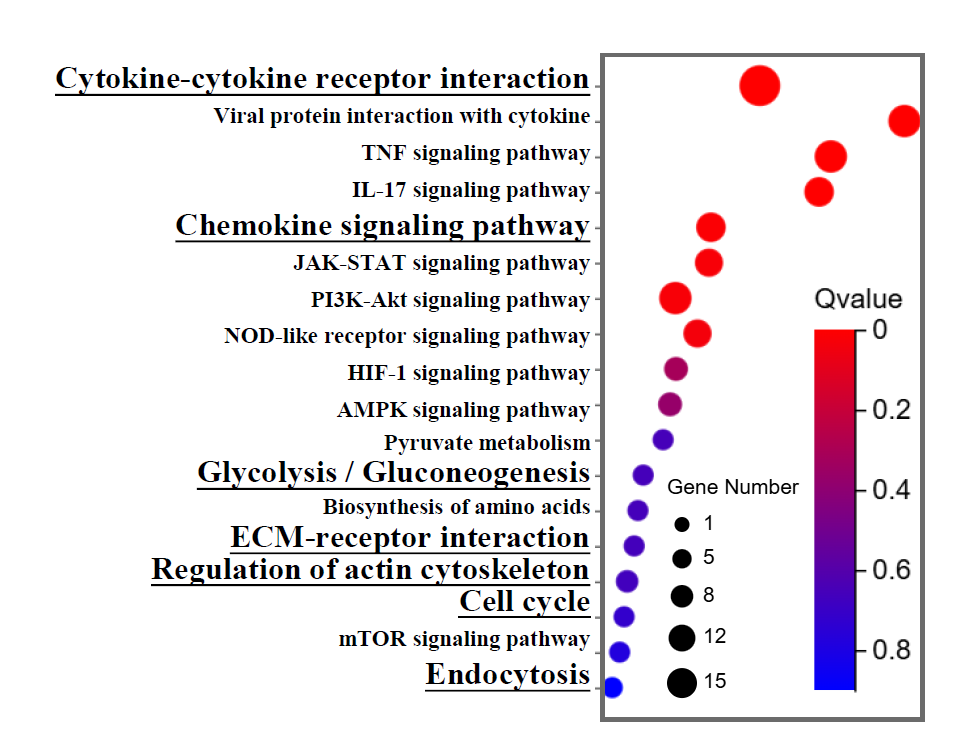


**Figure S1. KEGG Enrichment Analysis of Differentially Expressed Genes.**


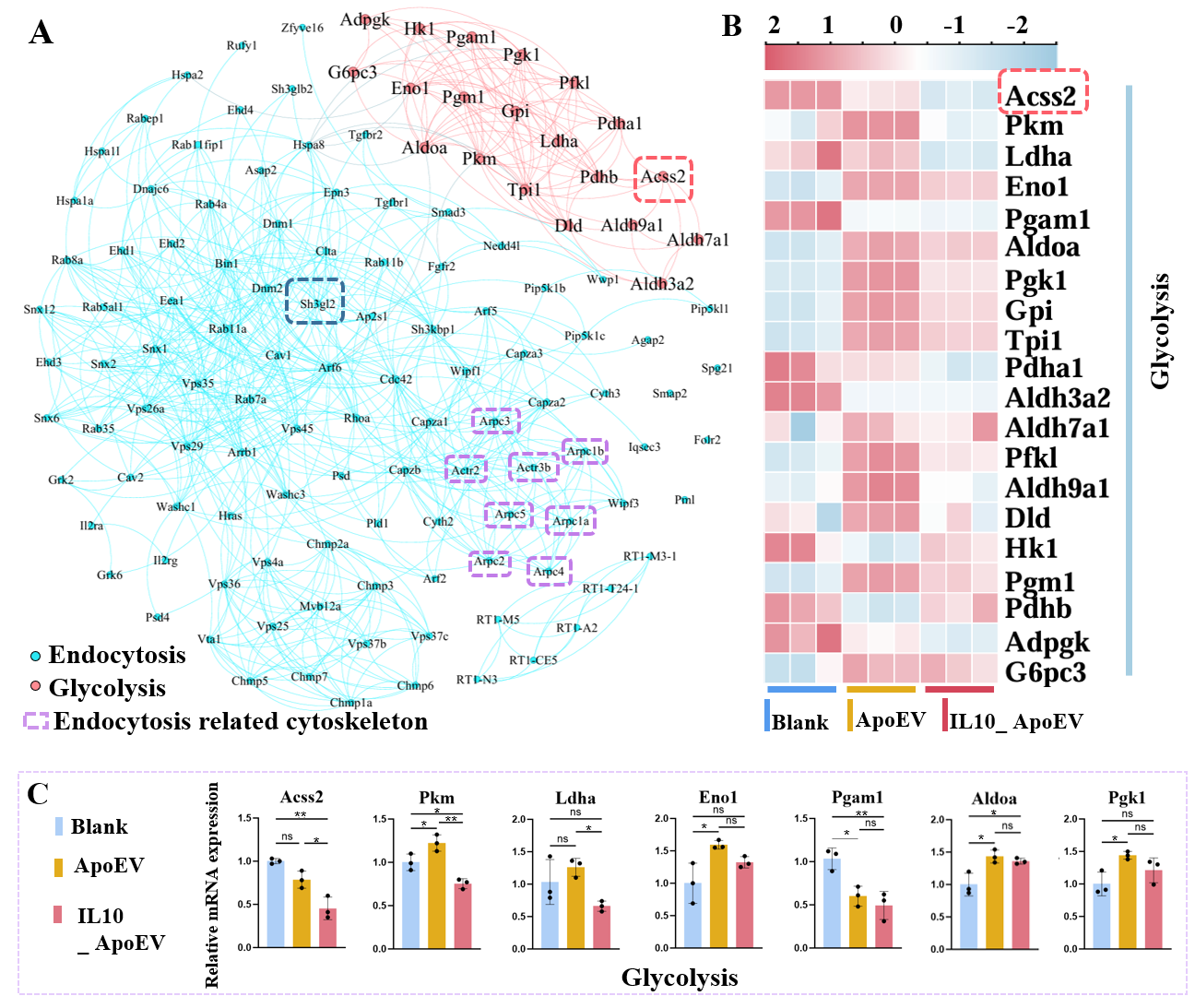


**Figure S2. Crosstalk Between Endocytosis, Cytoskeletal Remodeling, and Glycolysis in Fibroblasts Treated with IL10_ApoEV.** (A) Protein–protein interaction (PPI) network depicting the functional interplay among endocytosis-related genes, cytoskeletal regulators, and glycolytic enzymes. (B) Heatmap showing the differential expression of glycolysis-associated genes across the Blank, ApoEV, and IL10_ApoEV groups. (C) RT-qPCR validation of representative glycolytic genes (*Acss2*, *Pkm*, *Ldha*, *Eno1*, *Pgam1*, *Aldoa*, *Pgk1*).


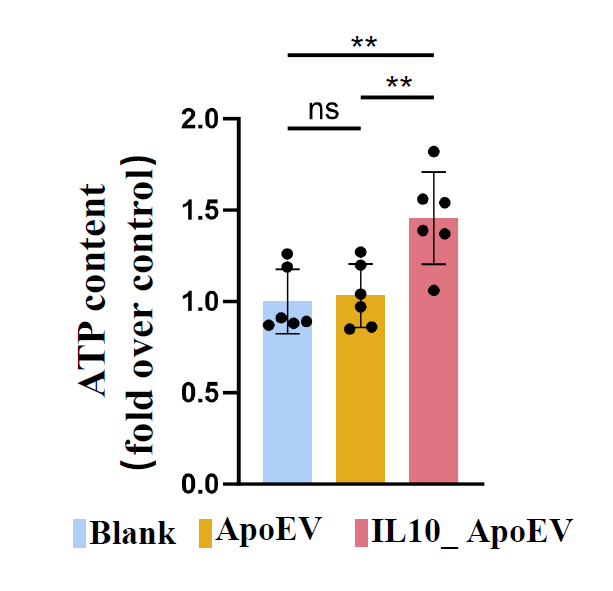


**Figure S3.** IL10_ApoEV Enhances Intracellular ATP Production in Fibroblasts.


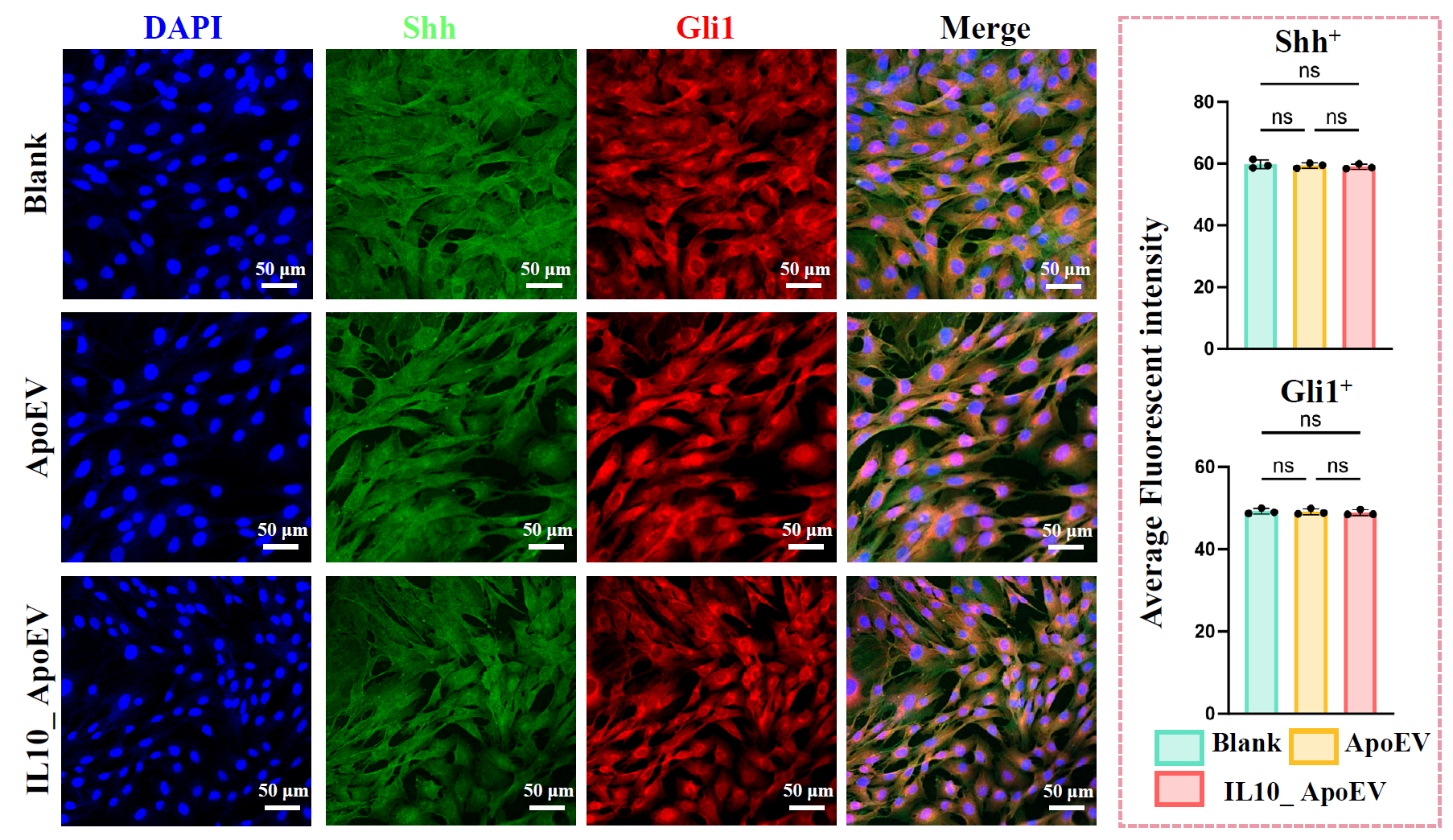


**Figure S4**. **Inhibition of Mitochondrial OXPHOS Reverses the Suppressive Effect of IL10_ApoEV on Hedgehog Signaling.** Representative immunofluorescence staining images of Shh (green) and Gli1 (red) in fibroblasts treated with oligomycin, a mitochondrial oxidative phosphorylation (OXPHOS) inhibitor, across Blank, ApoEV, and IL10_ApoEV groups. DAPI (blue) labels nuclei. Quantitative fluorescence analysis shows no significant differences in Shh^+^ and Gli1^+^ signal intensities between the groups after oligomycin treatment


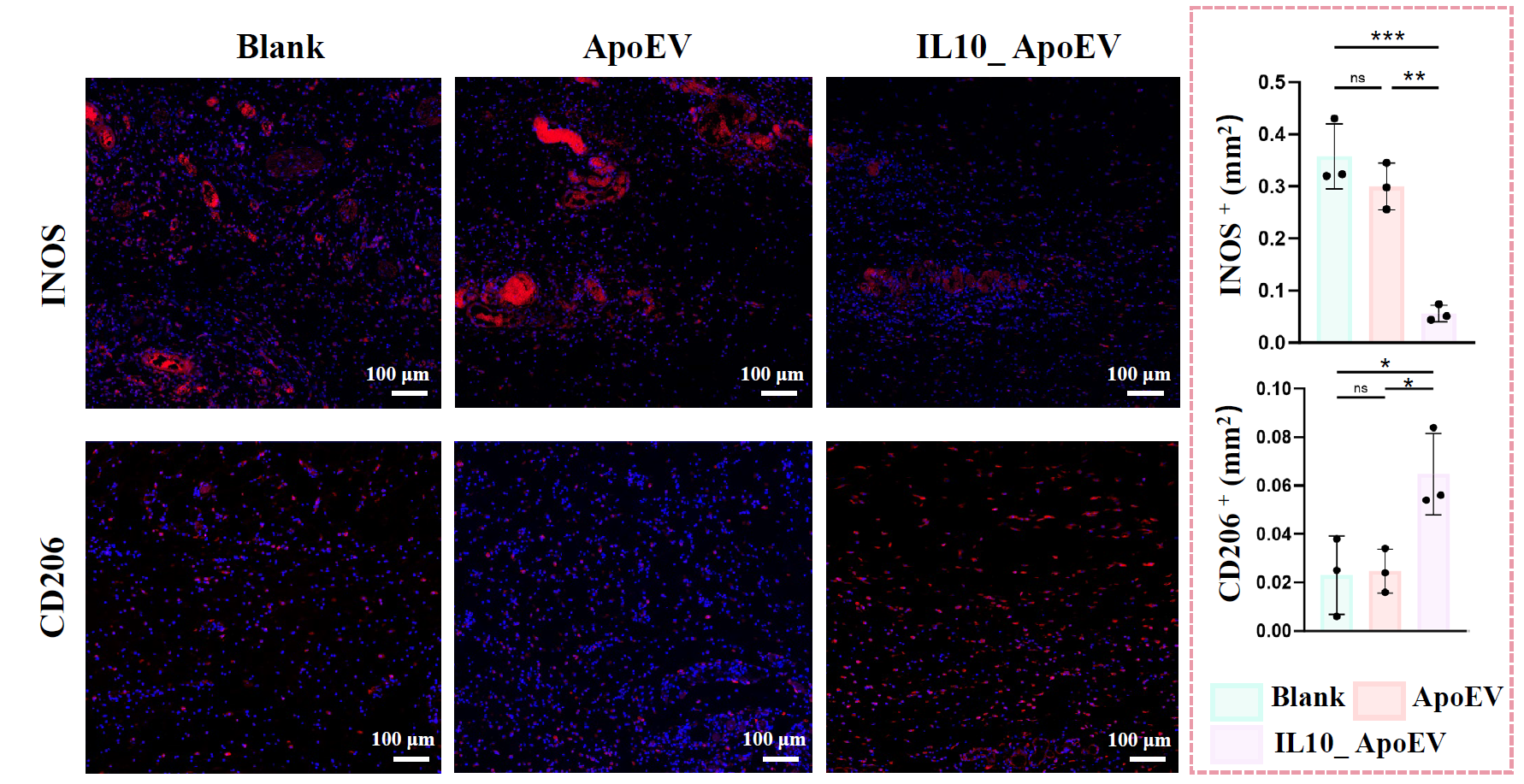


**Figure S5**. **Immunofluorescence staining and semi-quantitative analysis of macrophage polarization markers following collagen scaffold implantation**. Representative images show the expression of iNOS (a marker for pro-inflammatory M1 macrophages) and CD206 (a marker for anti-inflammatory M2 macrophages) in tissue sections 3 days post-implantation. Quantitative analysis of fluorescence intensity indicates that IL10_ApoEV treatment significantly reduces the presence of iNOS^+^ cells while increasing CD206^+^ cells

**Supplementary Table**

**Table S1**. **Primer sequences for fibroblasts**

| **Gene** | **Primer sequences** |  |
| --- | --- | --- |
| *Sh3gl2* | | Forward: 5’-TCTCGCATCCCATCATGTCG-3’  Reverse: 5’-CGTCTCCAGTGGCAAACTCT-3’ |
| *Folr2* | | Forward: 5’-AACAGAAGGCTTGCAGATGGT-3’  Reverse: 5’-AGGTCCAGTCCCAGCCTTTA-3’ |
| *Wipf3* | | Forward: 5’-CGTGATTGGCCGCACA-3’  Reverse: 5’-AATCCTCGAAGGACCAGCCT-3’ |
| *Cav2* | | Forward: 5’-GCTTCGAGGATCTGATCGCA-3’  Reverse: 5’-GCAAAAGCACTCCAAAGCGA-3’ |
| *Capza3* | | Forward: 5’-TTGACTGGCAACGGATCCTC-3’  Reverse: 5’- AGCACTTTGGCTTGAGACGA-3’ |
| *RT1-M5* | | Forward: 5’-CTACCTGAAGGGCGAGTGTG-3’  Reverse: 5’-CTACCTGAAGGGCGAGTGTG-3’ |
| *Arpc3* | | Forward: 5’-CCATGGTCAGCATCGCATTG-3’  Reverse: 5’-CGAGAGCTTTCCCGGTAGAC-3’ |
| *Arpc1a* | | Forward: 5’-GATCGCACTGAGCCCCAATA-3’  Reverse: 5’-TTCAGCTCGTGAGCTTTCGT-3’ |
| *Arpc5* | | Forward: 5’-GGGATGTCGAAGAACACGGT-3’  Reverse: 5’-GTAGGCATGAGTCCACCTCG-3’ |
| *Arpc1b* | | Forward: 5’-GAGACCCGAGGTCCAGGAG-3’  Reverse: 5’-CGTGCACCTTGTTCCACTTG-3’ |
| *Actr2* | | Forward: 5’-GAGGCGTCCTTCAGGTTTGA-3’  Reverse: 5’-ACGTTCCTCAAGGCATTGGT-3’ |
| *Arpc4* | | Forward: 5’-TCCTCAAGAACTTTTAGACCATCT-3’  Reverse: 5’-GTGACTCCAGGACACCTTCG -3’ |
| *Acss2* | | Forward: 5’-ATGTCCAGATGTCCAGATCTCCT-3’  Reverse: 5’-GTCCACAATGCTCCACAAGC-3’ |
| *Pkm* | | Forward: 5’- TACGCCCGAGGATCTCAGAA-3’  Reverse: 5’- TTCAGCCGAGCCACATTCAT-3’ |
| *Eno1* | | Forward: 5’-CTACTGCCAGTTCTAGAAGCCGTT-3’  Reverse: 5’-GTGAAGTTTTCTTCCCCAGCAG-3’ |
| *Pgam1* | | Forward: 5’-TACGGGGCATTGTCAAGCAT-3’  Reverse: 5’-CAGCTTCCATGGCTTTACGC-3’ |
| *Ldha* | | Forward: 5’-GAGCTGTGGTTGGTCCAGTT-3’  Reverse:5’-GCAGTTGGCAGTGTGTCTTG-3’ |
| *Aldoa* | | Forward: 5’- CTTCTTTCACTGCACCACAGG-3’  Reverse: 5’- GTTCTCCTCGGTGTTCTCGG-3’ |
| *Pgk1* | | Forward: 5’-TCCGCATCTCCATTTGGTGT-3’  Reverse: 5’-CCCGATGCAGTAAAGACGAG-3’ |
| *Cox8b* | | Forward: 5’-TCTCTTCCAAGCCAGCCAAA-3’  Reverse: 5’- ATGCTGCGGAGCTTCTCTTA-3’ |
| *Cox6b2* | | Forward: 5’-CCAGCTACGTTGGTCCACTT-3’  Reverse: 5’-GTAGCAGTTACGCGTCTGGT-3’ |
| *Cox10* | | Forward: 5’-GCTGCACCAGTGCGTTATG-3’  Reverse: 5’-TCGTCCTACTGAGCCTGTGA-3’ |
| *Atp6v1e1* | | Forward: 5’-CCTCGAATGATTGTGCGCTG-3’  Reverse: 5’-GGCTATTAGGTCCAGTCGGC-3’ |
| *Cox5a* | | Forward: 5’-ATGCTCGCTGGGTGACATAC-3’  Reverse: 5’-AGATGCGAACAGCACTAGCA-3’ |
| *Cycs* | | Forward: 5’-TTGTTGGACAGCCCCGATTTA-3’  Reverse: 5’-GCACGGGTGAGTCTTCTTGT-3’ |
| *Ndufa7* | | Forward: 5’-TGTGACCCTGAGTTGGCTAC-3’  Reverse: 5’-ACTCTCCCCAGAAGGAATCCA-3’ |
| *Uqcr10* | | Forward: 5’-GCAGACGCGGTCTACGATTA-3’  Reverse: 5’-AGTCTGTTTGCACGGTGGAG-3’ |
| *Mrpl43* | | Forward: 5’-AGTCGGTGGAGGAGATCACA-3’  Reverse: 5’-GTTTGTTGGTGAAGGGGTGC-3’ |
| *Mrpl36* | | Forward: 5’-GTCGATTGCCGATTTCTCCG-3’  Reverse: 5’-TCGCTGTTTATGCTTGGGGT-3’ |
| *Mrpl18* | | Forward: 5’-AAACGTGGTGGCTTGTGAGA-3’  Reverse: 5’-GGTGGCTGTAGATGGTCGTT-3’ |
| *Mrpl44* | | Forward: 5’-GTTCTTGCCATGGCTTCTGC-3’  Reverse: 5’-CCCTTCTTCACTCCCCGAAC-3’ |
| *Mrpl49* | | Forward: 5’-CCCAGAAACCGCAGGCTATT-3’  Reverse:5’-TTGTCACCTAGGATCAGGGC-3’ |
| *Cpt1a* | | Forward: 5’-CCTACCACGGCTGGATGTTT-3’  Reverse: 5’-TACAACATGGGCTTCCGACC-3’ |
| *Shh* | | Forward: 5’-GTAACGCTACGAGAGGAGGC-3’  Reverse: 5’-GAGCACCCGGTTGATGAGAA-3’ |
| *Dhh* | | Forward: 5’-TCGAGTCCACACCTTGCTTC-3’  Reverse:5’-GCTCCCTTGGTAAGTCCCAC-3’ |
| *Hhatl* | | Forward: 5’-CCCTCTCAGCCTCTTCAAGC -3’  Reverse: 5’-GGCCACATAGAGGACTACGC-3’ |
| *Ihh* | | Forward: 5’-AACTCGTGCCTCTTGCCTAC-3’  Reverse: 5’-AGTTCAGACGGTCCTTGCAG-3’ |
| *Gli1* | | Forward: 5’-AGAAGGACTTTCTGGTCTGCC-3’  Reverse:5’-ATGGAGAGATGGCCGTAGGA-3’ |
| *Hhip* | | Forward: 5’-TATCTCGGCCCTCAGTGTGA-3’  Reverse: 5’-AAGTCGCAGGAGTGTGTAGC-3’ |
| *Ccnd1* | | Forward: 5’-TCAAGTGTGACCCGGACTG-3’  Reverse: 5’-TGGGATCGATGTTCTGCTGG-3’ |
| *En2* | | Forward: 5’-GTGAAGAGGCGGTTTGGGAT-3’  Reverse:5’-GTGAAGAGGCGGTTTGGGAT-3’ |
| *Itga10* | | Forward: 5’-AGCATCACCCACGCCTATTC-3’  Reverse: 5’-AAACATCCCCCTTTCGGTCA-3’ |
| *Itgb6* | | Forward: 5’-ACCAGCAGTGTCTCTGCCTA-3’  Reverse: 5’-AACCATTCTGCGGTCACCAT-3’ |
| *Itga11* | | Forward: 5’-GGAGAAGGTGATCCGGCAAA-3’  Reverse: 5’-CTTGTTGGTGCCTTCCAAGC-3’ |
| *Col1a2* | | Forward: 5’-GAAATGGCAACTCAGCTCGC-3’  Reverse: 5’-GAAATGGCAACTCAGCTCGC-3’ |
| *Col4a6* | | Forward: 5’-CCAAATGCCACCCAGTTGTG-3’  Reverse: 5’-CTCAGAATGCAGGGAGCCAA-3’ |
| *Col1a1* | | Forward: 5’-ACATGTTCAGCTTTGTGGACC-3’  Reverse: 5’-CTTTGCATAGCACGCCATCG-3’ |
